# The bee family Halictidae (Hymenoptera, Apoidea) from Central Asia collected by the Kyushu and Shimane Universities Expeditions

**DOI:** 10.3897/BDJ.5.e15050

**Published:** 2017-10-20

**Authors:** Ryuki Murao, Osamu Tadauchi, Ryoichi Miyanaga

**Affiliations:** 1 Regional Environmental Planning Co., Ltd., Fukuoka, Japan; 2 Kyushu University, Fukuoka, Japan; 3 Faculty of Life and Environmental Science, Shimane University, Matsue, Japan

**Keywords:** Hymenoptera, Apoidea, Halictidae, Central Asia, Kazakhstan, Kyrgyzstan, Uzbekistan, Xinjiang Uyghur of China

## Abstract

**Background:**

Central Asia is one of the important centers of bee diversity in the Palearctic Region. However, there is insufficient information for many taxa in the central Asian bee fauna. The Kyushu and Shimane Universities (Japan) Expeditions to Kazakhstan, Kyrgyzstan, Uzbekistan, and Xinjiang Uyghur of China were conducted in the years 2000 to 2004 and 2012 to 2014.

**New information:**

Eighty-eight species of the bee family Halictidae Thomson, 1869 are enumerated including new localities in central Asia. *Halictus tibialis* Walker, 1871, *H. persephone* Ebmer, 1976, *Lasioglossum denislucum* (Strand, 1909), *L. griseolum* (Morawitz, 1872), *L. melanopus* (Dalla Torre, 1896), *L. nitidiusculum* (Kirby, 1802), *L. sexnotatulum* (Nylander, 1852), *L. subequestre* (Blüthgen, 1931), *L. sublaterale* (Blüthgen, 1931), and *L. zonulum* (Smith, 1848) are recorded from central Asia for the first time. Thirty-two species are newly recorded from Kazakhstan, 19 spp. from Kyrgyzstan, 2 spp. from Uzbekistan, and 11 spp. from Xinjiang Uyghur of China. The genus *Lasioglossum* dominated the number of species and individuals in the collection. The halictid fauna mostly composed of western to central Asian elements in our surveyed area.

## Introduction

Central Asia is a warm-temperate arid region located in the central part of the Eurasia Continent. It is sometimes referred to as Turkestan. In modern contexts, Central Asia includes the countries such as Kazakhstan, Kyrgyzstan, Tajikistan, Turkmenistan, Uzbekistan. Bees are generally considered to have higher diversity in the warm-temperate arid region than those in the tropics (Michener 1979). According to Michener (2007), the bee fauna is particularly rich in the Mediterranean basin and thence eastward to Central Asia in the Palearctic Region.

In central Asia and the western part of China, we conducted field surveys from 2000 to 2004 and 2012 to 2014, for the purpose of both taxonomic and biological studies of central Asian bees (Tadauchi 2005). A total of approximately 30,000 bee specimens were collected through this central Asian expedition. The present paper is the eleventh one of the series treating the result of this expedition (Tadauchi et al. 2005; Tadauchi 2006, Tadauchi 2008; Miyanaga et al. 2006; Mitai and Tadauchi 2008; Kuhlmann 2009; Shebl and Tadauchi 2009; Williams 2011; Mitai 2012; Murao et al. 2015). In the present paper, we report the collection data of the family Halictidae Thomson, 1869 except for the cleptoparasitic genus *Sphecodes* Latreille, 1804, with new locality data. We also discuss the faunal features of central Asian halictid bees in our surveyed area.

Halictidae is the second largest group of bees, with approximately 4,400 recognized species worldwide (Ascher and Pickering 2017). This family is found on all continents except for Antarctica. Four subfamilies are recognized (Michener 2007): Rophitinae Schenck, 1866, Nomiinae Robertson, 1904, Nomioidinae Börner, 1919, and Halictinae Thomson, 1869. Both morphological and molecular data support the monophyly of these four subfamilies (Pesenko 1999; Danforth et al. 2004). Halictid bees nest in the soil or rarely in rotting wood. They have a diverse social structure such as solitary, communal, semisocial, and eusocial (e.g., Michener 1974; Schwarz et al. 2007). Several genera and species are cleptoparasites or social parasites in nests of halictid or other bees. Most halictid species are known to be host-plant generalists except for some host-plant specialist taxa (e.g., the subfamily Rophitinae and *Lipotriches* Gerstaecker, 1858). In the temperate area of the world, halictid bees are common, ofen dominating other bees in number of individuals and species (Michener 2007).

The halictid bees from central Asia are mainly recorded by the following various researchers: Astafurova (2004), Astafurova and Pesenko (2005), Blüthgen (1923c), Blüthgen (1923a), Blüthgen (1923b), Blüthgen (1924), Blüthgen (1925), Blüthgen (1929), Blüthgen (1931), Blüthgen (1933a), Blüthgen (1933b), Blüthgen (1934b), Blüthgen (1934a), Blüthgen (1936), Blüthgen (1955), Ebmer (1972), Ebmer (1980), Ebmer (1995), Handlirsch (1888), Mitai (2012), Morawitz (1876), Morawitz (1880), Morawitz (1893), Morawitz (1894), Pallas (1773), Pèrez (1903), Pesenko (1979), Pesenko (1983), Pesenko (1984a), Pesenko (1984b), Pesenko (1984c), Pesenko (1985), Pesenko (1986), Pesenko (2005b), Pesenko (2005a), Pesenko (2006), Pesenko and Astafurova (2006), Pesenko and Wu (1997), Popov (1934), Popov (1935), Popov (1949), Popov (1952), Popov (1956), Popov (1958), Radoszkowski (1893), Strand (1909), Vachal (1902), Warncke (1976), Wu (1985). According to the database of Ascher and Pickering (2017), 219 species are listed from central Asia: 107 spp. from Kazakhstan, 66 spp. from Kyrgyzstan, 85 spp. from Tajikistan, 96 spp. from Turkmenistan, and 76 spp. from Uzbekistan.

## Materials and methods

The field survey was conducted in Kazakhstan (late May, 2000; late August to early September, 2002; late May to middle June, 2003; late April to late May, 2004), Kyrgyzstan (late May, 2000; middle to late August, 2003; early to late May, 2013; late August to early September, 2013; early to late June, 2014), Uzbekistan (late August to early September, 2012), and Xinjiang Uyghur Autonomous Region of China (late August, 2002). The collecting data and locality code are listed as follows:

**China**

CN1: East of Jeminay, alt. 1,080–1,300 m, Altay Prov., Xinjiang Uyghur Aut. Reg., N47°16'59.999", E86°00'59.999", 28. viii. 2002

CN2: Jeminay County, alt. 800–1,050 m, Altay Prov., Xinjiang Uyghur Aut. Reg., 27. viii. 2002

CN3: Fukang, alt. 520 m, Changji Prov., Xinjiang Uyghur Aut. Reg., 22. viii. 2002

CN4: Gaoquan, Kuitun city, Ili Prov., Xinjiang Urghur Aut. Reg., 26. viii. 2002

CN5: Guozigou, alt. 1,230 m, Ili Prov., Xinjiang Uyghur Aut. Reg., 25. viii. 2002

CN6: Jinghe, alt. 540 m, Ili Prov., Xinjiang Uyghur Aut. Reg., 24. viii. 2002

CN7: Kuitun City, alt. 530 m, Ili Prov., Xinjiang Uyghur Aut. Reg., 24. viii. 2002

CN8: near Sayram Lake, alt. 1,970 m, Ili Prov., Xinjiang Uyghur Aut. Reg., 25. viii. 2002 (Fig. 1a, b)

CN9: Northwest of Kuitun, alt. 450 m, Ili Prov., Xinjiang Uyghur Aut. Reg., 26. viii. 2002

CN10: Qingshuihe, alt. 780 m, Ili Prov., Xinjiang Uyghur Aut. Reg., 25. viii. 2002

CN11: West of Kuitun, alt. 560 m, Ili Prov., Xinjiang Uyghur Aut. Reg., N44°25'59.999", E83°57'59.999", 26. viii. 2002

CN12: Yining city, Ili Prov., Xinjiang Uyghur Aut. Reg., 25. viii. 2002

CN13: Sugongta, Turpan Prov., Xinjiang Uyghur Aut. Reg., 23. viii. 2002

**Kazakhstan**

KZ1: Almaty city, 24. v. 2000, 29. v. 2000, 31. viii. 2002

KZ2: Botanical garden, Almaty, 25. v. 2003

KZ3: Degeres, alt. 850 m, Almaty, 28. v. 2000

KZ4: Fabrichini, alt. 850 m, Almaty, 26. v. 2000

KZ5: Kemertogan, Almaty, 26. v. 2000

KZ6: Koktobe, Almaty, 21. v. 2004

KZ7: Kurday, alt. 800–880 m, Almaty, 26–28. v. 2000

KZ8: Medew, Almaty, 24. v. 2000, 21. v. 2004

KZ9: National Museum, Almaty, 25. v. 2003

KZ10: Nogaibay, alt. 780 m, Almaty, 28. v. 2000

KZ11: Panpilof PK., Almaty, 25. v. 2000

KZ12: Uzenagash, Almaty, 28. v. 2000

KZ13: Big Almaty Lake, alt. 1,230–2,050 m, Almaty Prov., 31. viii–1. ix. 2002, 19. vi. 2003, 22. v. 2004, 28. viii. 2004 (Fig. 1c)

KZ14: Chilik riverside, East of Almaty, Almaty Prov., 2–3. ix. 2002

KZ15: East of Almaty, Almaty Prov., 2. ix. 2002

KZ16: Riverside Ili river, Northwest of Kapchagay, Almaty Prov., 18–19. v. 2004

KZ17: South of Almaty, alt. 1,580 m, Almaty Prov., 31. viii. 2002

KZ18: Akkol-Talas, Jambyl Prov., 14. v. 2004

KZ19: Alga, near Koradai, Jambyl Prov., 18. vi. 2003

KZ20: Berkaza Valley, 60km, Southwest of Karatau City, Jambyl Prov., 12. v. 2004

KZ21: East of Taraz, alt. 570–600 m, Jambyl Prov., N42°58'59.999", E73°24'59.999", 3. ix. 2002, 8. ix. 2002

KZ22: Jambyl Prov., alt. 703 m, N43°06'54.699", E74°42'22.299", 5. v. 2013

kZ23: Karatau City, alt. 600 m, Jambyl Prov., 13. v. 2004

KZ24: Karatau-Janatas, alt. 680 m, Jambyl Prov., 13. v. 2004

KZ25: Kenen, near Otar, Jambyl Prov., 20. viii. 2003

KZ26: Kordai, alt. 540–1,080 m, Jambyl Prov., 3. ix. 2002, 17. v. 2004, 27. viii. 2004

KZ27: Moyenkum-Chu, Jambyl Prov., 17. v. 2004

KZ28: Muyunkum-Kumozek, alt. 325 m, Muyunkum desert, Jambyl Prov., 16. v. 2004

KZ29: near Taraz, alt. 540–600 m, Jambyl Prov., 3. ix. 2002

KZ30: North of Janatas, alt. 420 m, Jambyl Prov., 13. v. 2004

KZ31: Northwest of Akkol, Muyunkum desert, Jambyl Prov., 14. v. 2004

KZ32: Northwest of Tatti, alt. 325–562 m, Muyunkum desert, Jambyl Prov., 15. v. 2004

KZ33: Riverside Chu river, Moyenkum, alt. 480 m, Jambyl Prov., 17. v. 2004

KZ34: South of Muyunkum, alt. 406 m, Muyunkum desert, Jambyl Prov., 15. v. 2004

KZ35: Achisai, alt. 500–700 m, Mts. Karatau, South Kazakhstan Prov., 3–6. vi. 2003

KZ36: Akbasutau, South Kazakhstan Prov., 10. vi. 2003

KZ37: Aksu Jabagly, alt. 1,080–1,830 m, South Kazakhstan Prov., 1–3. v. 2004, 3. ix. 2002, 4. ix. 2002, 7. ix. 2002, 8. v. 2004, 11. v. 2004, 11. vi. 2003, 13. vi. 2003, 14. vi. 2003, 27. v. 2003, 28. v. 2003 (Fig. 1d, e)

KZ38: Aksu valley, Jabagly, alt. 130–560 m, South Kazakhstan Prov., 6. ix. 2002, 16. vi. 2003 (Fig. 1f)

KZ39: Baijansai, alt. 660–1,030 m, Mts. Karatau, South Kazakhstan Prov., 9. vi. 2003

KZ40: Boskhog village, alt. 226 m, North of Chordara, South Kazakhstan Prov., 1. v. 2004

KZ41: Chordara, alt. 200 m, West of Tashkent, South Kazakhstan Prov., 30. iv. 2004

KZ42: Daubaba, alt. 700–800 m, South Kazakhstan Prov., 13. vi. 2003

KZ43: East of Boroldy village, Mts. Karatau, South Kazakhstan Prov., 10. v. 2004

KZ44: East of Chimkent, alt. 570m, South Kazakhstan Prov., 30. v. 2003

KZ45: Eskara, East of Syrdarya river, South Kazakhstan Prov., 9. v. 2004

KZ46: Hot spring, West of Kamsomolskoe, Kyzylkum desert, South Kazakhstan Prov., 1–3. v. 2004

KZ47: Janatas, South Kazakhstan Prov., 7. vi. 2003

KZ48: Jarekbas, near Shayan, South Kazakhstan Prov., 8–10. vi. 2003

KZ49: Kamsomolskoe, North of Chordara, South Kazakhstan Prov., 1. v. 2003, 1. v. 2004

KZ50: Kantagi, alt. 550–700 m, near Kentau, Mts. Karatau, South Kazakhstan Prov., 1–2. vi. 2003

KZ51: Karaalma alt. 1,210 m, near Jabagly, South Kazakhstan Prov., 7. ix. 2002, 17. vi. 2003

KZ52: Karamola, Kyzylkum desert, South Kazakhstan Prov., 6. v. 2004

KZ53: Kenestobe, near Shayan, South Kazakhstan Prov., 8. vi. 2003

KZ54: Kogam, alt. 250 m, near Otrar, South Kazakhstan Prov., 31. v. 2003

KZ55: Kyzylkum desert, South Kazakhstan Prov., 2. v. 2004

KZ56: Lake Charbarinskoe, Chordara, alt. 180 m, West of Tashkent, South Kazakhstan Prov., 30. iv. 2004

KZ57: National border, Chernjaevka, South Kazakhstan Prov., 26. v. 2003

KZ58: North of Boroldy village, Mts. Karatau, South Kazakhstan Prov., 10. v. 2004

KZ59: North of Chimkent, alt. 400 m, South Kazakhstan Prov., 30. v. 2003

KZ60: Plain North of Karamola, Kyzylkum desert, South Kazakhstan Prov., 4. v. 2004 (Fig. 2a)

KZ61: Polevod, riverside Syrdarya river, South Kazakhstan Prov., 7–9. v. 2004

KZ62: Seslavino, alt. 960 m, Daubaba river, South Kazakhstan Prov., 11–13. vi. 2003

KZ63: Shayan-Birlik, South Kazakhstan Prov., 8. vi. 2003

KZ64: Sutkent village, North of Kamsomolskoe, South Kazakhstan Prov., 3. v. 2004

KZ65: Togusken, semi-desert, near Janatas, South Kazakhstan Prov., 7. vi. 2003

KZ66: Ulken-Kaindy, Jabagly, alt. 1,090–2,000 m, South Kazakhstan Prov., 4–5. ix. 2002, 15. vi. 2003

KZ67: West of Chimkent, South Kazakhstan Prov., 29. iv. 2004

KZ68: West of Kamsomolskoe, Kyzylkum desert, South Kazakhstan Prov., 1–2. v. 2004

KZ69: Nurly village, 3. ix. 2002

**Kyrgyzstan**

KG1: Bishkek City, 27–28. v. 2000

KG2: Kemin, alt. 1,000 m, near Bishkek, 23. viii. 2003

KG3: Ara Archa, Chuy Prov., alt. 1,700–2,152 m, N42°58'59.999", E73°24'59.999", 21. viii. 2003, 6. v. 2013, 22. v. 2013, 31. viii. 2013, 5. v. 2014, 5. vi. 2014, 21. vi. 2014 (Fig. 2b)

KG4: Don-Aryk, Chuy Prov., alt. 1,027 m, N42°44'29.199", E75°12'00.799", 23. vi. 2014

KG5: Issyk-Ata, Chuy Prov., alt. 950–1,875 m, N42°35'58.099", E74°54'24.599", 27. v. 2000, 22. viii. 2003, 14–15. v. 2013, 27. viii. 2013 (Fig. 2c)

KG6: Koi Tash, Chuy Prov., alt. 1,256–2,091 m, N42°41'16.899", E74°40'23.899", 23. v. 2013, 25. viii. 2013, 6. vi. 2014, 22. vi. 2014 (Fig. 2d)

KG7: Krasnaya Rechka, Chuy Prov., alt. 782–827 m, N42°51'27.099", E74°59'21.999", 13. v. 2013, 16. v. 2013

KG8: near Dzhar-Bashy, Chuy Prov., alt. 936m, N42°45'51.899", E75°00'22.099", 27. viii. 2013

KG9: near Issyk-Ata, Chuy Prov., alt. 1,167–1,339 m, N42°41'19.999", E75°03'06.899", 13. v. 2013

KG10: near Jany-Alysh, Chuy Prov., alt. 1,000–1,018 m, N42°49'15.899", E75°33'57.899", 28. viii. 2013, 1. ix. 2013

KG11: near Jil-Aryk, Chuy Prov., alt. 1,055 m, N42°45'22.199", E75°48'25.299", 1. ix. 2013

KG12: near Kageti, Chuy Prov., alt. 1,100–1,313 m, N42°42'41.999", E75°07'59.599", 23. vi. 2014

KG13: near Kemin, Chuy Prov., alt. 1,263–1,348 m, N42°41'20.999", E75°52'48.099", 17. v. 2013

KG14: near Tagetan National Park, Chuy Prov., alt. 1,515 m, N42°37'11.399", E75°08'11.199", 23. vi. 2014

KG15: Tagetan National Park, Chuy Prov., alt. 1,756 m, N42°33'53.999", E75°07'13.999", 23. vi. 2014

KG16: Aksuu, alt. 2,000 m, near Karakol, East of Lake Issyk-Kul, Issyk-Kul Prov., 25. viii. 2004

KG17: Arashan, alt. 1,850–1,900 m, near Karakol, East of Lake Issyk-Kul, Issyk-Kul Prov., 25. viii. 2004

KG18: Barskoon, Issyk-Kul Prov., alt. 1,864 m, N42°07'12.399", E77°35'11.599", 10. v. 2013

KG19: Chon Ak Suu, Issyk-Kul Prov., alt. 1,700–1,991 m, N42°46'06.199", E77°28'30.199", 8. v. 2013, 21. v. 2013, 24. viii. 2004, 28. viii. 2013

KG20: Jele Tobe, Issyk-Kul Prov., alt. 1,730 m, N42°26'53.199", E78°12'31.999", 17. vi. 2014

KG21: Jeti Oguz, Issyk-Kul Prov., alt. 2,048 m, N42°04'41.999", E77°35'43.699", 17. vi. 2014

KG22: Konstanchinofuka, Issyk-Kul Prov., alt. 1,784 m, N42°32'38.499", E78°39'44.699", 18. vi. 2014, 18. ix. 2014

KG23: near Balykchy, Issyk- Kul Prov., alt. 1,754 m, N42°20'31.299", E76°05'00.899", 20. v. 2013

KG24: near Balykchy, Issyk-Kul Prov., alt. 1,632m, N42°29'25.099", E76°22'18.399", 7. v. 2013

KG25: near Barskoon, Issyk-Kul Prov., alt. 2,048 m, N42°04'41.999", E77°35'43.699", 17. vi. 2014

KG26: near Barskoon, Issyk-Kul Prov., alt. 2,387m, N41°59'47.699", E77°37'18.399", 10. v. 2013

KG27: near Bokonbayevo, Issyk-Kul Prov., alt. 1,798–1,841 m, N42°08'20.599", E77°00'59.999", 11. v. 2013

KG28: near Chychkan, Issyk-Kul Prov., alt. 1,656 m, N42°17'29.399", E77°49'18.399", 19. vi. 2014

KG29: near San Tash, Issyk-Kul Prov., alt. 1,862 m, N42°44'24.900", E78°48'24.599", 9. v. 2013 (Fig. 2e)

KG30: near Semenovskoye, National Park., Issyk-Kul Prov., alt. 1,818–1,860 m, N42°44'46.799", E77°32'43.599", 29. viii. 2013

KG31: near Tilekmat, Issyk-Kul Prov., alt. 1,707 m, N42°24'34.799", E78°06'53.899", 17. vi. 2014

KG32: Novovoznesenovka, Issyk-Kul Prov., alt. 1,798 m, N42°36'20.299", E78°46'44.299", 18. vi. 2014

KG33: Semenovka, alt. 1,700 m, North of Lake Issyk-Kul, Issyk-Kul Prov., 24. viii. 2004

KG34: Skiing ground, Karakol, Issyk-Kul Prov., alt. 2,240 m, N42°36'20.299", E78°46'44.299", 16. vi. 2014

KG35: Teploklyuchenka, Issyk-Kul Prov., alt. 1,802 m, N42°30'07.799", E78°30'17.599", 18. vi. 2014

KG36: Tilekmat, Issyk-Kul Prov., alt. 1,698 m, N42°25'44.499", E78°09'14.699", 17. vi. 2014

KG37: Tongu, Issky-Kul Prov., alt. 1,677 m, N42°08'52.899", E77°01'48.499", 19. vi. 2014

KG38: West of Kaji-Say, Issyk-Kul Prov., alt. 1,619 m, N42°09'26.299", E77°07'08.599", 19. vi. 2014

KG39: Ak-Kiya, Naryn Prov., alt. 1,850–1,879 m, N42°11'08.699", E75°42'26.499", 15. vi. 2014 (Fig. 2f)

KG40: Ak-Tal, Naryn Prov., alt. 1,635 m, N41°25'13.499", E75°01'58.299", 12. vi. 2014

KG41: Doron Pass, Naryn Prov., alt. 2,887 m, N41°49'59.499", E75°45'36.099", 15. vi. 2014

KG42: East of Naryn, Naryn Prov., alt. 2,280–2,333 m, N41°27'14.499", E76°21'33.199", 9. vi. 2014

KG43: Jangy-Talap, Naryn Prov., alt. 1,710–1,989 m, N42°32'55.599", E75°01'47.599", 12. vi. 2014, 14. vi. 2014

KG44: Kala Bulung, Naryn Prov., alt. 2,288 m, N41°05'02.499", E75°33'48.199", 13. vi. 2014

KG45: Kara-Suu, Naryn Prov., alt. 2,101–2,153 m, N41°07'58.899", E75°40'36.199", 2. ix. 2013, 13. vi. 2014

KG46: Kochikoru, Naryn Prov., alt. 1,849 m, N42°12'24.699", E75°47'07.399", 7. vi. 2014

KG47: Moldo-Ashuu Pass, Naryn Prov., alt. 2,218–2,947 m, N41°39'52.499", E75°01'27.799", 4. ix. 2013, 10–11. vi. 2014 (Fig. 3a)

KG48: Naryn, Naryn Prov., alt. 1,991–2,008 m, N41°25'58.099", E75°52'28.599", 19. v. 2013

KG49: Naryn, Naryn Prov., alt. 2,153–2,280 m, N41°26'52.099", E76°16'32.599", 18. v. 2013 (Fig. 3b)

KG50: near Alysh Park, Naryn Porv., alt. 2,227 m, N41°26'50.599", E76°15'09.599", 9. vi. 2014

KG51: near At-Bashi, Naryn Prov., alt. 2,117 m, N41°11'43.399", E75°49'38.099", 13. vi. 2014

KG52: West of Naryn, Naryn Prov., alt. 1,736–1,742 m, N41°23'42.999", E75°10'02.699", 12. vi. 2014 (Fig. 3c)

KG53: Krasnayarichika, alt. 800 m, 27. v. 2000

**Uzbekistan**

UZ1: Aydar Lake, 28. viii. 2012 (Fig. 3d)

UZ2: Botanical Garden, Tashkent, 1–4. ix. 2012

UZ3: Dalla Hovli, Northeast of Chirchik, 2. ix. 2012 (Fig. 3e)

UZ4: Dalla Hovli, West of Parkent, 1. ix. 2012

UZ5: Gijduvon, North of Bukhara, 28. viii. 2012

UZ6: Golbog, West of Parkent, 1. ix. 2012

UZ7: Gushrabot, South of Aydar Lake, 29. viii. 2012 (Fig. 3f)

UZ8: Madaniyat village, East of Samarkand, 31. viii. 2012 (Fig. 4a)

UZ9: Nurota, South of Aydar Lake, 29. viii. 2012

UZ10: Parkent, 1. ix. 2012

UZ11: Qorodaro river side, Samarkand, 30. viii. 2012

UZ12: South of Aydar Lake, 29. viii. 2012 (Fig. 4b)

UZ13: Samarkand～Tashkent, 1. ix. 2012 (Fig. 4c)

UZ14: Sardoba, West of Guliston, 31. viii. 2012

UZ15: Southwest of Yangiyo'l, South of Tashkent, 3. ix. 2012 (Fig. 4d)

All specimens are preserved in the Entomological Laboratory, Faculty of Agriculture, Kyushu University, Fukuoka, Japan. The specimens data are also accessible from Tadauchi and Murao (2009).

Identification of halictid bee specimens is based on the collection both Biologiezentrum/Oberösterreichisches Landesmuseum (Linz, Austria) and Zoological Institute, Russian Academy of Sciences (St. Petersburg, Russia), and the following keys: Astafurova and Pesenko (2005), Pesenko (2005b), Pesenko (2005c), Pesenko (2006), and Pesenko and Astafurova (2006).

Information on distribution for each species in the present paper is based on Astafurova and Pesenko (2005), Ebmer (1995), Ebmer (1997), Ebmer (2005), Ebmer and Sakagami (1985), Niu et al. (2005), Niu et al. (2007), Pesenko (2005b), Pesenko (2005a), Pesenko (2006), Pesenko and Astafurova (2006), Pesenko and Wu (1997), Pesenko et al. (2000), and Ascher and Pickering (2017).

## Checklists

### A list of halictid species collected by Central Asian Expeditions

#### Rophitinae

Schenck, 1866

#### Dufourea paradoxa atrocoerulea

(Morawitz, 1875)

##### Ecological interactions

###### Host of

Asteraceae sp.

##### Distribution

This subspecies is endemic in the Pamir Mountain area in central Asia.

#### Rophites (Rophitoides) canus

Eversmann, 1852

##### Ecological interactions

###### Host of

Apiaceae sp., *Brassica* sp., *Echium vulgare*, *Vicia villosa*

##### Distribution

Europe to eastern Asia. This species has been recorded from Kyrgyzstan, Turkmenistan, Uzbekistan, and Xinjiang Uyghur of China in central Asia.

#### Systropha (Systropha) curvicornis

(Scopoli, 1770)

##### Ecological interactions

###### Host of

Asteraceae sp.

##### Distribution

Europe to northwestern China.

#### Nomiinae

Robertson, 1904

#### Pseudapis (Nomiapis) diversipes

(Latreille, 1806)

##### Ecological interactions

###### Host of

*Achillea* sp., *Brassica* sp., *Cruciferae* sp., Lamiaceae sp., Leguminosae sp., Melilotus officinalis subsp. suaveolens, *Mentha asiatica*, *Solidago* sp., *Tamarix* sp., *Vicia villosa*.

##### Distribution

Europe, north Africa to eastern Asia. This species has been recorded from Kazakhstan, Kyrgyzstan, Turkmenistan, Uzbekistan, and Xinjiang Uyghur of China in central Asia.

#### Pseudapis (Nomiapis) femoralis

(Pallas, 1773)

##### Ecological interactions

###### Host of

*Brassica* sp., *Echinops* sp.

##### Distribution

Europe to eastern Asia. This species has been recorded from Kazakhstan and Xinjiang Uyghur of China in central Asia.

##### Notes

New record for Kyrgyzstan.

#### Pseudapis (Nomiapis) fugax

(Morawitz, 1877)

##### Ecological interactions

###### Host of

*Cirsium* sp., Lamiaceae sp., *Tamarix* sp.

##### Distribution

Europe and north Africa to eastern Asia. This species has been recorded from Kazakhstan, Tajikistan, Turkmenistan, Uzbekistan, and Xinjiang Uyghur of China in central Asia.

#### Nomioidinae

Börner, 1919

#### Ceylalictus (Ceylalictus) variegatus

(Olivier, 1789)

##### Ecological interactions

###### Host of

*Peganum harmala*, *Tamarix* sp.

##### Distribution

Palearctic to the northern Oriental Region. This species has been recorded from Kazakhstan and Kyrgyzstan in central Asia.

##### Notes

New records for Uzbekistan and Xinjiang Uyghur of China.

#### Nomioides gussakovskiji

Blüthgen, 1933

##### Ecological interactions

###### Host of

*Tamarix* sp.

##### Distribution

Western to eastern Asia. This species has been recorded from Kazakhstan, Tajikistan, Turkmenistan, Uzbekistan, and Xinjiang Uyghur of China in central Asia.

#### Nomioides ino

(Nurse, 1904)

##### Ecological interactions

###### Host of

*Chondrilla* sp., *Tamarix* sp.

##### Distribution

Western to eastern Asia. This species is recorded from Kazakhstan, Tajikistan, Turkmenistan, and Uzbekistan in central Asia.

##### Notes

New record for Xinjiang Uyghur of China.

#### Nomioides minutissimus minutissimus

(Rossi, 1790)

##### Ecological interactions

###### Host of

Asteraceae sp., *Chondrilla* sp., Lamiaceae sp., Leguminosae sp., *Peganum harmala*, *Tamarix* sp.

##### Distribution

Europe to eastern Asia. The nominotypical subspecies has been recorded from Kazakhstan, Tajikistan, and Uzbekistan in central Asia.

##### Notes

New records for Kyrgyzstan and Xinjiang Uyghur of China.

#### Halictinae

Thomson, 1869

#### Halictus (Argalictus) senilis

(Eversmann, 1852)

##### Ecological interactions

###### Host of

*Tamarix* sp.

##### Distribution

Europe, north Africa to eastern Asia. This species has been recorded from Kazakhstan, Kyrgyzstan, Turkmenista, Uzbekistan, and Xinjiang Uyghur of China in central Asia.

#### Halictus (Argalictus) tibialis

Walker, 1871

##### Ecological interactions

###### Host of

*Mentha asiatica*.

##### Distribution

Middle East.

##### Notes

New record for central Asia (Kazakhstan).

#### Halictus (Halictus) brunnescens

(Eversmann, 1852)

##### Ecological interactions

###### Host of

*Achillea biebersteinii*, *Achillea* sp., *Allium sativum*, *Althaea rosea*, Apiaceae sp., *Aster canescens*, Asteraceae sp., *Brassica* sp., Caprifoliaceae sp., *Chondrilla* sp., *Cichorium intybus*, *Cirsium* sp., *Cruciferae* sp., *Echinops ritro*, *Echinops* sp., Fabaceae sp., *Ferula tenuisecta*, *Geranium* sp., *Hibiscus* sp., Lamiaceae sp., Leguminosae sp., *Mentha asiatica*, *Origanum tyttanthum*, Polygonaceae sp., Rosaceae sp., *Salix* sp., *Schrenkia golickeana*, *Tamarix ramosissima*, *Tamarix* sp., *Taraxacum* sp., *Trifolium repens*, *Umbelliferae* sp., *Vicia* sp., *Vicia villosa*.

##### Distribution

Europe, north Africa to eastern Asia. This species has been recorded from Kazakhstan, Kyrgyzstan, Turkmenista, Uzbekistan, and Xinjiang Uyghur of Chia in central Asia.

#### Halictus (Halictus) duplocinctus

Vachal, 1902

##### Ecological interactions

###### Host of

Asteraceae sp., Gentianaceae sp., *Mentha asiatica*, *Tamarix* sp., *Taraxacum* sp.

##### Distribution

Middle East to central Asia. This species has been recorded from Kazakhstan, Kyrgyzstan, Tajikistan, Turkmenista, Uzbekistan, and Xinjiang Uyghur of China in central Asia.

#### Halictus (Halictus) quadricinctus

(Fabricius, 1776)

##### Ecological interactions

###### Host of

*Achillea* sp., Asteraceae sp., *Brassica* sp., *Cirsium* sp., Fabaceae sp., Rosaceae sp., *Trifolium repens*, *Vicia villosa*.

##### Distribution

Europe to eastern Asia. This species has been recorded from Kazakhstan, Kyrgyzstan, Tajikistan, Uzbekistan, and Xinjiang Uyghur of China in central Asia.

#### Halictus (Hexataenites) resurgens

Nurse, 1903

##### Ecological interactions

###### Host of

*Achillea biebersteinii*, *Achillea* sp., *Aster canescens*, Asteraceae sp., *Brassica* sp., *Breea setosa*, *Chondrilla* sp., *Chrysanthemum* sp., *Cichorium intybus*, *Cirsium* sp., *Dahlia* sp., Geraniaceae sp., Lamiaceae sp., *Mentha asiatica*, *Origanum tyttanthum*, *Papaver pavoninum*, *Rosa kokanica*, *Sysimbrium* sp., *Tagetes* sp., *Tamarix ramosissima*, *Tamarix* sp., *Taraxacum* sp., *Trifolium repens*.

##### Distribution

Southern Europe, northeastern Africa to central Asia. This species has been recorded from Kazakhstan, Kyrgyzstan, Tajikistan, Turkmenistan, Uzbekistan, and Xinjiang Uyghur of China.

#### Halictus (Monilapis) compressus transvolgensis

Pesenko, 1985

##### Ecological interactions

###### Host of

*Achillea biebersteinii*, *Achillea* sp., Apiaceae sp., *Aster canescens*, Asteraceae sp., *Brassica juncea*, *Brassica* sp., *Breea setosa*, *Chondrilla* sp., *Cicerbita azurea*, *Cichorium intybus*, *Cirsium* sp., *Eremurus cristatus*, Fabaceae sp., *Ferula tenuisecta*, Melilotus officinalis subsp. suaveolens, *Mentha asiatica*, *Origanum tyttanthum*, *Rhamnus cathartica*, *Rosa kokanica*, Rosaceae sp., *Taraxacum* sp., *Trifolium repens*, *Trollius altaicus*, *Vicia villosa*.

##### Distribution

Central to eastern Asia. This subspecies has been recorded from Kazakhstan, Kyrgyzstan, and Xinjiang Uyghur of China in central Asia.

#### Halictus (Mucoreohalictus) indefinitus

Blüthgen, 1923

##### Ecological interactions

###### Host of

*Achillea biebersteinii*, *Chondrilla* sp.

##### Distribution

North Africa to eastern Asia. This species has been recorded Kazakhstan, Tajikistan, and Turkmenistan in central Asia.

##### Notes

New record for Xinjiang Uyghur of China.

#### Halictus (Mucoreohalictus) mucidus

Blüthgen, 1923

##### Ecological interactions

###### Host of

*Achillea biebersteinii*, *Aster canescens*, Asteraceae sp., *Chondrilla* sp., *Cichorium intybus*, *Cirsium* sp., *Ferula tenuisecta*, *Mentha asiatica*, *Taraxacum* sp.

##### Distribution

Afghanistan and Tajikistan.

##### Notes

New record for Kazakhstan.

#### Halictus (Mucoreohalictus) mucoreus

Eversmann, 1852

##### Ecological interactions

###### Host of

*Achillea biebersteinii*, *Achillea* sp., Apiaceae sp., Asteraceae sp., *Brassica juncea*, *Brassica* sp., Fabaceae sp., *Halimodendron holodendron*, Leguminosae sp., Melilotus officinalis subsp. suaveolens, *Peganum harmala*, *Tamarix* sp., *Trifolium pretense*, *Trifolium repens*, *Vicia villosa*.

##### Distribution

South Europe to central Asia. This species has been recorded from Turkmenistan and Xinjiang Uyghur of China in central Asia.

##### Notes

New records for Kazakhstan, Kyrgyzstan, and Uzbekistan.

#### Halictus (Mucoreohalictus) pollinosus cariniventris

Morawitz, 1876

##### Ecological interactions

###### Host of

*Brassica* sp., *Mentha asiatica*.

##### Distribution

Europe, north Africa to eastern Asia. This subspecies has been recorded from Kyrgyzstan, Tajikistanm Uzbekistan, and Xinjiang Uyghur of Chia in central Asia.

##### Notes

New record for Kazakhstan.

#### Halictus (Mucoreohalictus) pseudomucoreus

Ebmer, 1975

##### Distribution

Western to central Asia. This species has been recorded from Turkmenistan and Xinjiang Uyghur of China in central Asia.

##### Notes

New record for Kyrgyzstan.

#### Halictus (Placidohalictus) bulbiceps

Blüthgen, 1929

##### Ecological interactions

###### Host of

*Tamarix* sp.

##### Distribution

Central Asia. This species has been recorded from Kazakhstan.

##### Notes

New record for Xinjiang Uyghur of Chia.

#### Halictus (Placidohalictus) fuscicollis

Morawitz, 1876

##### Distribution

Middle East to central Asia (Turkestan).

##### Notes

New record for Xinjiang Uyghur of China.

#### Halictus (Platyhalictus) alfkenellus cedens

Blüthgen, 1931

##### Ecological interactions

###### Host of

*Brassica* sp., *Vicia villosa*.

##### Distribution

Europe to central Asia. This subspecies has been recorded from Kazakhstan and Turkmenistan in central Asia.

##### Notes

New record for Kyrgyzstan.

#### Halictus (Platyhalictus) minor

Morawitz, 1876

##### Ecological interactions

###### Host of

*Aster canescens*, *Cichorium intybus*, *Cirsium* sp., Cruciferae sp., *Ferula tenuisecta*, Melilotus officinalis subsp. suaveolens, *Mentha asiatica*, *Serophularia* sp., *Tamarix* sp., *Taraxacum* sp.

##### Distribution

Western to eastern Asia. This species has been recorded from Kazakhstan, Kyrgyzstan, Tajikistan, Turkmenista, Uzbekistan, and Xinjiang Uyghur of China in central Asia.

#### Halictus (Platyhalictus) takuiricus

Blüthgen, 1936

##### Ecological interactions

###### Host of

Melilotus officinalis subsp. suaveolens.

##### Distribution

Middle East to central Asia. This species has been recorded from Kazakhstan, Kyrgyzstan, Tajikistan, Turkmenista, and Xinjiang Uyghur of China in central Asia.

#### Halictus (Protohalictus) bucharicus

Blüthgen, 1936

##### Ecological interactions

###### Host of

*Ferula tenuisecta*.

##### Distribution

Central Asia (Kazakhstan and Tajikistan).

#### Halictus (Protohalictus) rubicundus

(Christ, 1791)

##### Ecological interactions

###### Host of

Fabaceae sp., *Spiraea* sp.

##### Distribution

Holarctic. This species has been recorded from Kazakhstan, Kyrgyzstan, and Xinjiang Uyghur of China in central Asia.

#### Halictus (Seladonia) leucaheneus leucaheneus

Ebmer, 1972

##### Distribution

Europe to eastern Asia. This nominotypical subspecies has been recorded from Kazakhstan, Kyrgyzstan, and Xinjiang Uyghur of China in central Asia.

#### Halictus (Seladonia) pjalmensis pjalmensis

Strand, 1909

##### Ecological interactions

###### Host of

*Achillea biebersteinii*, *Achillea* sp., *Allium sativum*, Apiaceae sp., *Aster canescens*, Asteraceae sp., *Brassica juncea*, *Brassica* sp., Brassicaceae sp., *Breea setosa*, *Chondrilla* sp., *Cichorium intybus*, *Erigeron* sp., *Halimodendron holodendron*, Leguminosae sp., Lythraceae sp., *Mentha asiatica*, *Potentilla* sp., Rosaceae sp., *Tamarix* sp., *Taraxacum* sp., *Trifolium repens*, *Umbelliferae* sp., *Vicia villosa*.

##### Distribution

Central to eastern Asia. This nominotypical subspecies has been recorded from Kazakhstan and Xinjiang Uyghur of China in central Asia.

##### Notes

This subspecie is newly recorded from Kyrgyzstan in this study.

#### Halictus (Seladonia) seladonius

(Fabricius, 1794)

##### Ecological interactions

###### Host of

*Achillea biebersteinii, Achillea* sp., *Apiaceae* sp., *Aster canescens*, *Aster* sp., *Asteraceae* sp., *Brassica* sp., *Chondrilla* sp., *Chrysanthemum* sp., *Cicerbita azurea*, *Cosmos bipinnatus*, *Cruciferae* sp., *Echium vulgare*, Melilotus officinalis subsp. suaveolens, *Mentha asiatica*, *Schrenkia golickeana*, *Tamarix* sp., *Trifolium repens*, *Umbelliferae* sp.

##### Distribution

Europe, north Africa to central Asia. This species has been recorded from Kyrgyzstan, Tajikistan, and Uzbekistan in central Asia.

##### Horizon

New record for Kazakhstan.

#### Halictus (Seladonia) transbaikalensis

Blüthgen, 1933

##### Distribution

Eastern Asia.

##### Notes

New record for central Asia (Kazakhstan).

#### Halictus (Tytthalictus) maculatus maculatus

Smith, 1848

##### Ecological interactions

###### Host of

*Brassica* sp., Convolvulaceae sp.

##### Distribution

Europe to eastern Siberia. The nominotypical subspecies has been recorded from Kazakhstan, Turkmenistan, and Xinjiang Uyghur of China in central Asia.

##### Notes

New record for Kyrgyzstan.

#### Halictus (Tytthalictus) palustris

Morawitz, 1876

##### Ecological interactions

###### Host of

*Achillea* sp., *Allium sativum*, *Aster canescens*, Asteraceae sp., *Brassica* sp., *Cichorium intybus*, *Cirsium* sp., *Ferula tenuisecta*, *Labiatae* sp., Melilotus officinalis subsp. suaveolens, *Mentha asiatica*, *Potentilla* sp., Rosaceae sp., *Taraxacum* sp. Umbelliferae sp.

##### Distribution

Central Asia. This species has been recorded from Kazakhstan, Kyrgyzstan, Tajikistan, Uzbekistan, and Xinjiang Uyghur of China.

#### Halictus (Vestitohalictus) nasica

Morawitz, 1876

##### Ecological interactions

###### Host of

*Tamarix* sp.

##### Distribution

North Africa to central Asia. This species has been recorded from Turkmenistan in central Asia.

##### Notes

New record for Kazakhstan.

#### Halictus (Vestitohalictus) persephone

Ebmer, 1976

##### Distribution

Europe to north Africa.

##### Notes

New record for central Asia (Kazakhstan).

#### Halictus (Vestitohalictus) pulvereus

Morawitz, 1874

##### Ecological interactions

###### Host of

*Achillea biebersteinii*, Asteraceae sp., *Chondrilla* sp., *Tamarix* sp., *Trifolium repens*.

##### Distribution

Southern Europe, north Africa to eastern Asia. This species has been recorded from Turkmenistan, Uzbekistan, and Xinjiang Uyghur of China in central Asia.

##### Notes

New record for Kazakhstan.

#### Lasioglossum (Dialictus) alanum

(Blüthgen, 1929)

##### Ecological interactions

###### Host of

*Taraxacum* sp.

##### Distribution

Middle East to central Asia.

##### Notes

New records for Kazakhstan and Kyrgyzstan.

#### Lasioglossum (Dialictus) fedtschenkoi

(Blüthgen, 1937)

##### Distribution

Western to central Asia. This species has been recorded from Kyrgyzstan in central Asia.

##### Notes

New record for Kazakhstan.

#### Lasioglossum (Dialictus) smeathmanellum

(Kirby, 1802)

##### Ecological interactions

###### Host of

*Mentha asiatica*, *Taraxacum* sp.

##### Distribution

Europe.

##### Notes

New record for central Asia (Kazakhstan and Kyrgyzstan).

#### Lasioglossum (Hemihalictus) buccale

(Pérez, 1903)

##### Ecological interactions

###### Host of

*Chondrilla* sp.

##### Distribution

Europe to central Asia. This species has been recorded from Kazakhstan, Kygyzstan, Tajikistan, and Uzbekistan in central Asia.

#### Lasioglossum (Hemihalictus) ciscapum

(Blüthgen, 1931)

##### Distribution

Western to central Asia. This species has been recorded from Kazakhstan, Kyrgyzstan, and Uzbekistan in central Asia.

#### Lasioglossum (Hemihalictus) clypeare

(Schenck, 1853)

##### Distribution

Europe, north Africa to central Asia. This species has been recorded from Kyrgyzstan in central Asia.

##### Notes

New record for Kazakhstan.

#### Lasioglossum (Hemihalictus) clypeiferellum

(Strand, 1909)

##### Ecological interactions

###### Host of

*Achillea* sp.

##### Distribution

Europe, north Africa to eastern Asia. This species has been recorded from Tajikistan in central Asia.

##### Notes

New record for Kyrgyzstan.

#### Lasioglossum (Hemihalictus) croceipes

(Morawitz, 1876)

##### Ecological interactions

###### Host of

*Achillea biebersteinii*, *Achillea* sp., *Cirsium* sp., *Convolvulus arvensis*, *Ferula tenuisecta*, *Ixioliron tataricum*, *Schrenkia golickeana*.

##### Distribution

Central Asia (Turkestan).

##### Notes

New records for Kazakhstan and Kyrgyzstan.

#### Lasioglossum (Hemihalictus) denislucum

(Strand, 1909)

##### Ecological interactions

###### Host of

*Achillea biebersteinii*, *Brassica* sp., Brassicaceae sp., *Trifolium repens*.

##### Distribution

Europe to western Asia.

##### Notes

New record for central Asia (Kazakhstan and Kyrgyzstan).

#### Lasioglossum (Hemihalictus) griseolum

(Morawitz, 1872)

##### Ecological interactions

###### Host of

*Achillea* sp., *Bidens* sp., *Brassica* sp.

##### Distribution

Europe, north Africa to western Asia.

##### Notes

New record for central Asia (Kazakhstan and Kyrgyzstan).

#### Lasioglossum (Hemihalictus) laevinode

(Morawitz, 1876)

##### Ecological interactions

###### Host of

*Aster canescens*, *Ferula tenuisecta*.

##### Distribution

Middle East to central Asia. This species has been recorded from Kyrgyzstan in central Asia.

##### Notes

New record for Kazakhstan.

#### Lasioglossum (Hemihalictus) limbellum

(Morawitz, 1876)

##### Distribution

Europe, north Africa to eastern Asia. This species has been recorded from Uzbekistan in central Asia. It is newly recorded from Kazakhstan.

##### Notes

New record for Kazakhstan.

#### Lasioglossum (Hemihalictus) longirostre

(Morawitz, 1876)

##### Ecological interactions

###### Host of

*Cirsium* sp., *Ferula tenuisecta*, *Ixioliron tataricum*, *Origanum tyttanthum*, *Taraxacum* sp.

##### Distribution

Middle East to central Asia. This species has been recorded from Kazakhstan and Kyrgyzstan in central Asia.

#### Lasioglossum (Hemihalictus) lucidulum

(Schenck, 1861)

##### Ecological interactions

###### Host of

*Achillea* sp., *Brassica* sp.

##### Distribution

Europe, north Africa to eastern Asia. This species has been recorded from Kazakhstan, Kyrgyzstan, and Turkmenistan in central Asia.

#### Lasioglossum (Hemihalictus) matianense pluto

Ebmer, 1980

##### Ecological interactions

###### Host of

*Aconitum* sp., Caprifoliaceae sp., *Eremurus cristatus*, Fabaceae sp., *Potentilla* sp., Rosaceae sp., *Salix* sp., *Spiraea* sp., *Taraxacum* sp.

##### Distribution

Central to eastern Asia. This subspecies has been recorded from Kazakhstan, Kyrgyzstan, and Uzbekistan in central Asia.

#### Lasioglossum (Hemihalictus) melanopus

(Dalla Torre, 1896)

##### Distribution

Middle East.

##### Notes

New record for central Asia (Kazakhstan and Kyrgyzstan).

#### Lasioglossum (Hemihalictus) nitidiusculum

(Kirby, 1802)

##### Distribution

Europe, north Africa to Middle East.

##### Notes

New record for central Asia (Kazakhstan).

#### Lasioglossum (Hemihalictus) persicum

(Cockerell, 1919)

##### Distribution

Western to central Asia. This species has been recorded from Kazakhstan, Kyrgyzstan, Turkmenistan, and Uzbekistan in central Asia.

#### Lasioglossum (Hemihalictus) popovi

(Blütkahgen, 1931)

##### Distribution

Central Asia (Kyrgyzstan and Uzbekistan).

#### Lasioglossum (Hemihalictus) pseudonigripes

(Blüthgen, 1934)

##### Ecological interactions

###### Host of

Apiaceae sp., Boraginaceae sp., *Brassica* sp., Brassicaceae sp., Fabaceae sp., Rosaceae sp., *Spiraea* sp., *Tamarix* sp., *Taraxacum* sp.

##### Distribution

Western to eastern Asia. This species has been recorded from Kyrgyzstan in central Asia.

##### Notes

New record for Kazakhstan.

#### Lasioglossum (Hemihalictus) subaenescens asiaticum

(Dalla Torre, 1896)

##### Ecological interactions

###### Host of

Umbelliferae sp.

##### Distribution

Western to eastern Asia. This subspecies has been recorded from Kazakhstan, Kyrgyzstan, Tajikistan, Turkmenistan, Uzbekistan, and Xinjiang Uyghur of China in central Asia.

#### Lasioglossum (Hemihalictus) tschardschuicum

(Blüthgen, 1931)

##### Distribution

Central Asia (Uzbekistan).

##### Notes

New record for Kazakhstan.

#### Lasioglossum (Hemihalictus) villosulum

(Kirby, 1802)

##### Ecological interactions

###### Host of

*Tamarix* sp.

##### Distribution

Widely distributed from Plearctic to Oriental Region. This species has been recorded from Kyrgyzstan in central Asia.

##### Notes

New record for Kazakhstan.

#### Laioglossum (Lasioglossum) acephalum

(Blüthgen, 1923)

##### Distribution

Central Asia (Turkestan).

##### Notes

This species may be newly recorded from Kazakhstan in this study.

#### Lasioglossum (Lasioglossum) costulatum

(Kriechbaumer, 1873)

##### Ecological interactions

###### Host of

Asteraceae sp., *Brassica juncea*, *Ferula tenuisecta*, *Geranium* sp., *Mentha asiatica*, *Trifolium repens*.

##### Distribution

Europe, north Africa to central Asia. This species has been recorded from Kazakhstan in central Asia.

##### Notes

New record for Kyrgyzstan.

#### Lasioglossum (Lasioglossum) equestre

(Morawitz, 1876)

##### Ecological interactions

###### Host of

*Achillea* sp., *Brassica* sp., *Echinops* sp., Fabaceae sp., Leguminosae sp., *Rhamnus cathartica*, *Taraxacum* sp., *Trifolium repens*, Umbelliferae sp.

##### Distribution

Central Asia (Kazakhstan and Kyrgyzstan).

#### Lasioglossum (Lasioglossum) fulvitarse

(Morawitz, 1876)

##### Ecological interactions

###### Host of

*Salix* sp., *Spiraea* sp., *Taraxacum* sp.

##### Distribution

Middle East to central Asia (Turkestan).

##### Notes

This species may be newly recorded from Kazakhstan and Kyrgyzstan in this study.

#### Lasioglossum (Lasioglossum) lebedevi

Ebmer, 1972

##### Distribution

Western to central Asia (Turkestan).

##### Notes

This species may be newly recorded from Kazakhstan in this study.

#### Lasioglossum (Lasioglossum) quadrinotatiforme

Ebmer, 1980

##### Ecological interactions

###### Host of

*Brassica* sp., Brassicaceae sp., *Halimodendron holodendron*, Leguminosae sp., *Rosa kokanica*, *Taraxacum* sp.

##### Distribution

Central Asia (Tajikistan).

##### Notes

New records for Kazakhstan and Kyrgyzstan.

#### Lasioglossum (Lasioglossum) sexnotatulum

(Nylander, 1852)

##### Ecological interactions

###### Host of

*Spiraea* sp., *Taraxacum* sp.

##### Distribution

Europe.

##### Notes

New record for central Asia (Kyrgyzstan).

#### Lasioglossum (Lasioglossum) subequestre

(Blüthgen, 1931)

##### Ecological interactions

###### Host of

*Papaver rhoeas*.

##### Distribution

Middle East.

##### Notes

New record for central Asia (Kazakhstan).

#### Lasioglossum (Lasioglossum) sublaterale

(Blüthgen, 1931)

##### Distribution

Southern Asia.

##### Notes

New record for central Asia (Kazakhstan).

#### Lasiolglossum (Lasioglossum) verae

Pesenko, 1986

##### Ecological interactions

###### Host of

Asteraceae sp., *Melilotus suaveolens*.

##### Distribution

Central Asia (Kazakhstan).

##### Notes

New record for Xinjiang Uyghur of China.

#### Lasioglossum (Lasioglossum) xanthopus

(Kirby, 1802)

##### Ecological interactions

###### Host of

*Achillea biebersteinii*, *Achillea* sp., *Aconitum* sp., *Brassica* sp., Brassicaceae sp., *Cirsium* sp., *Eremurus cristatus*, *Ferula tenuisecta*, *Ixioliron tataricum*, Leguminosae sp., Melilotus officinalis subsp. suaveolens, *Potentilla* sp., *Rosa kokanica*, Rosaceae sp., *Taraxacum* sp., *Trifolium pretense*, *Trifolium repens*, *Vicia villosa*.

##### Distribution

Europe, north Africa to eastern Asia. This species has been recorded from Kazakhstan and Kyrgyzstan in central Asia.

#### Lasioglossum (Leuchalictus) discum

(Smith, 1853)

##### Ecological interactions

###### Host of

*Achillea biebersteinii*, *Achillea* sp., Asteraceae sp., *Brassica juncea*, *Brassica* sp., Brassicaceae sp., *Chondrilla* sp., *Chrysanthemum* sp., *Cichorium intybus*, *Cirsium* sp., Cruciferae sp., *Dahlia* sp., *Ferula tenuisecta*, *Halimodendron holodendron*, Lamiaceae sp., Leguminosae sp., Melilotus officinalis subsp. suaveolens, *Mentha asiatica*, *Tamarix* sp., *Trifolium repens*, Umbelliferae sp., *Vicia villosa*.

##### Distribution

Europe, north Africa to central Asia. This species has been recorded from Kazakhstan, Kyrgyzstan, Tajikistan, Turkmenistan, Uzbekistan, and Xinjiang Uyghur of China in central Asia.

#### Lasioglossum (Leuchalictus) leucozonium

(Schrank, 1781)

##### Ecological interactions

###### Host of

*Achillea* sp., Apiaceae sp., Asteraceae sp., *Brassica* sp., Brassicaceae sp., *Chondrilla* sp., *Cichorium intybus*, *Tamarix* sp., *Taraxacum* sp., *Trifolium repens*.

##### Distribution

Holarctic. This species has been recorded from Kyrgyastan and Uzbekistan in central Asia.

##### Notes

New records for Kazakhstan and Xinjiang Uyghur of China.

#### Lasioglossum (Leuchalictus) niveocinctum

(Blüthgen, 1923)

##### Distribution

Western to eastern Asia. This species has been recorded from Kazakhstan, Turkmenistan, and Uzbekistan in central Asia.

##### Notes

New record for Xinjiang Uyghur of China.

#### Lasioglossum (Leuchalictus) scutellare

(Morawitz, 1876)

##### Ecological interactions

###### Host of

Asteraceae sp., *Chondrilla* sp., *Convolvulus arvensis*, *Halimodendron holodendron*, *Tamarix* sp., *Taraxacum* sp., *Trifolium repens*.

##### Distribution

Central Asia (Kazakhstan, Tajikistan, Turkmenistan, and Uzbekistan).

##### Notes

New records for Kyrgyzstan and Xinjiang Uyghur of China.

#### Lasioglossum (Leuchalictus) zonulum

(Smith, 1848)

##### Ecological interactions

###### Host of

*Halimodendron holodendron*.

##### Distribution

Holarctic.

##### Notes

New record for central Asia (Kazakhstan).

#### Lasioglossum (Sphecodogastra) albipes albipes

(Fabricius, 1781)

##### Ecological interactions

###### Host of

*Taraxacum* sp.

##### Distribution

Europe to eastern Asia. This nominotypical subspecies has been recorded from Uzbekistan in central Asia.

##### Notes

New records for Kazakhstan and Kyrgyzstan.

#### Lasioglossum (Sphecodogastra) aprilinum

(Morawitz, 1876)

##### Ecological interactions

###### Host of

Apiaceae sp., Fabaceae sp., *Tamarix* sp.

##### Distribution

Central to eastern Asia. This species has been recorded from Kazakhstan and Uzbekistan in central Asia.

##### Notes

New records for Kyrgyzstan and Xinjiang Uyghur of China.

#### Lasioglossum (Sphecodogastra) calceatum

(Scopoli, 1763)

##### Ecological interactions

###### Host of

*Aster canescens*, Asteraceae sp., *Brassica* sp., Brassicaceae sp., *Chondrilla* sp., *Cicerbita azurea*, *Cichorium intybus*, *Cirsium* sp., *Crataegus* sp., Fabaceae sp., *Mentha asiatica*, *Origanum tyttanthum*, *Potentilla* sp., Ranunculaceae sp., *Salix* sp., *Spiraea* sp., *Tamarix* sp., *Taraxacum* sp., *Trifolium repens*, *Vicia villosa*.

##### Distribution

Europe to eastern Asia. This species has been recorded from Kazakhstan, Kyrgyzstan, Uzbekistan, and Xinjiang Uyghur of China in central Asia.

#### Lasioglossum (Sphecodogastra) cingulatum

(Morawitz, 1876)

##### Ecological interactions

###### Host of

*Brassica* sp., Leguminosae sp., Umbelliferae sp.

##### Distribution

Central Asia. This species has been recorded from Kazakhstan, Tajikistan, and Turkmenistan in central Asia.

##### Notes

New record for Kyrgyzstan.

#### Lasioglossum (Sphecodogastra) hyalinipenne

(Morawitz, 1876)

##### Ecological interactions

###### Host of

Cruciferae sp., *Spiraea* sp., *Taraxacum* sp.

##### Distribution

Middle East to central Asia. This species has been recorded from Kyrgyzstan, Tajikistan, and Uzbekistan in central Asia.

##### Notes

New record for Kazakhstan.

#### Lasioglossum (Sphecodogastra) obscuratum

(Morawitz, 1876)

##### Ecological interactions

###### Host of

*Aster canescens*, *Brassica juncea*, *Chondrilla* sp., *Mentha asiatica*, *Rosa kokanica*, *Taraxacum* sp.

##### Distribution

Europe to central Asia. This species has been recorded from Turkmenistan in central Asia.

##### Notes

New record for Kazakhstan.

#### Lasioglossum (Sphecodogastra) rhynchites

(Morawitz, 1876)

##### Ecological interactions

###### Host of

*Aconitum* sp., Apiaceae sp., *Aster canescens*, Asteraceae sp., Brassicaceae sp., *Breea setosa*, Caprifoliaceae sp., *Chondrilla* sp., *Cichorium intybus*, *Cirsium* sp., *Mentha asiatica*, *Spiraea* sp., *Sysimbrium* sp., *Taraxacum* sp., *Trifolium repens*, *Vicia villosa*.

##### Distribution

Western to central Asia. This species has been recorded from Kazakhstan, Kyrgyzstan, Turkmenistan, and Uzbekistan in central Asia.

#### Lasioglossum kozlovi

(Friese, 1914)

##### Ecological interactions

###### Host of

*Tamarix* sp.

##### Distribution

Central to eastern Asia. This species has been recorded from Turkestan and Xinjiang Uyghur of China in central Asia.

#### Lasioglossum mandibulare

(Morawitz, 1866)

##### Ecological interactions

###### Host of

*Halimodendron holodendron*, *Tamarix* sp.

##### Distribution

Europe to western Asia. This species has been recorded from central Asia (Kazakhstan and Xinjiang Uyghur of China).

#### Lasioglossum marginatum

(Brullé, 1832)

##### Ecological interactions

###### Host of

*Achillea biebersteinii*, *Achillea millefolium*, *Brassica juncea*, *Brassica* sp., Brassicaceae sp., Caprifoliaceae sp., *Cirsium* sp., Cruciferae sp., *Eremurus cristatus*, *Ferula tenuisecta*, *Hypericum scabrum*, *Ixioliron tataricum*, Leguminosae sp., *Medicago lupulima*, *Rhamnus cathartica*, *Rosa kokanica*, Rosaceae sp., *Salix* sp., *Schrenkia golickeana*, *Taraxacum* sp., *Trifolium pretense*, *Trifolium repens*, *Trollius altaicus*, Umbelliferae sp.

##### Distribution

Europe, north Africa to southern Asia. This species has been recorded Kazakhstan, Kyrgyzstan, and Uzbekistan in central Asia.

#### Lasioglossum salinaecola

(Friese, 1916)

##### Distribution

Middle East to central Asia (Turkestan).

##### Notes

New record for Kazakhstan.

## Discussion

A total of 88 species belonging to eight genera in four subfamilies were collected during our survey. We found 10 new records for Central Asia, 32 new records for Kazakhstan, 19 new records for Kyrgyzstan, two new records for Uzbekistan, and 11 new records for Xinjiang Uyghur of China. The subfamily Halictinae dominated the bee fauna both in the number of species (78 / 88 spp.) and individuals (15968 / 16384 exs.) (Table 1; Suppl. material 1). Particularly, the genus *Lasioglossum* was the most common group (50 / 88 spp.; 13220 / 16384 exs.). This genus is known to dominate both in the number of species and individuals in warm-temparate regions (Sakagami and Fukuda 1973; Maeta et al. 2003). This tendency is similar in our surveyed area of central Asia.

Based on Pesenko et al. (2000), the relative abundance of halictid bees in our surveyed area are shown as follows.

1) Common and mass species (over 1,800 exs.), 1 sp.: *Lasioglossum marginatum* (10,257 exs.).

2) Common species (251–1,800 exs.), 6 spp.: *Halictus brunnescens*, *H. compressus transvolgensis*, *H. mucoreus*, *H. pjalmensis pjalmensis* (Fig. 6c), *Lasioglossum calceatum*, and *L. xanthopus*.

3) Relatively common species (41–250 exs.), 24 spp.: *Pseudapis diversipes* (Fig. 5d), *Ceylalictus variegatus* (Fig. 6a), *Nomioides minutissimus minutissimus* (Fig. 6b), *Halictus mucidus*, *H. minor*, *H. palustris*, *H. pulvereus*, *H. quadricinctus*, *H. resurgens*, *H. seladonius*, *H. senilis*, *Lasioglossum aprilinum*, *L. clypeiferellum*, *L. croceipes*, *L. discum*, *L. equestre*, *L. hyalinipennis*, *L. leucozonium*, *L. longirostre*, *L. matianense pluto*, *L. pseudonigripes*, *L. quadrinotatiforme*, *L. rhynchites*, and *L. scutellare*.

4) Relatively rare and uncommon species (8–40 exs.), 25 spp.: *Rophites canus* (Fig. 5b), *Pseudapis fugax*, *Nomioides gussakouskiji*, *Halictus bucharicus*, *H. duplocinctus*, *H. indefinitus*, *H. nasica*, *H. rubicundus*, *Lasioglossum acephalum*, *L. alanum*, *L. albipes albipes*, *L. cingulatum*, *L. costulatum* (Fig. 6d), *L. denislucum*, *L. fulvitarse*, *L. kozlovi*, *L. laevinode*, *L. lucidulum*, *L. mandibulare*, *L. melanopus*, *L. obscuratm*, *L. popovi*, *L. sexnotatulum*, *L. subaenescens asiaticum*, and *L. subequestre*.

5) Rare species (1–7 exs.), 32 spp.: *Dufourea paradoxa atrocoerulea* (Fig. 5a), *Systropha curvicornis* (Fig. 5c), *Pseudapis femoralis*, *Nomioides ino*, *Halictus alfkenellus cedens*, *H. bulbiceps*, *H. fuscicollis*, *H. leucaheneus leucaheneus*, *H. maculatus maculatus*, *H. persephone*, *H. pollinosa cariniventris*, *H. pseudomucoreus*, *H. takuiricus*, *H. tibialis*, *H. transbaikalensis*, *Lasioglossum buccale*, *L. ciscapum*, *L. clypeale*, *L. fedtschenkoi*, *L. griseolum*, *L. lebedevi*, *L. limbellum limbellum*, *L. nitidiusculum*, *L. niveocinctum*, *L. persicum*, *L. salinaecola*, *L. smeathmanellum*, *L. sublaterale*, *L. tschardschuicum*, *L. verae*, *L. villosulum*, and *L. zonulum*.

The most dominant species in individuals was *Lasioglossum marginatum* occurring mainly in the Western Palearctic Region. One of the reasons, it seems that *L. marginatum* is known as a eusocial species having the largest colony-size (worker number exceeds 400 individuals for per colony) in the eusocial *Lasioglossum* (Plateaux-Quénu 1962; Michener 1974).

The distribution of each species was roughly classified into seven elements as follows:

1) Holarctic, widely distributed from Palearctic to Nearctic Region (3 spp.): *Halictus rubicundus*, *Lasioglossum leucozonium*, and *L. zonulum*.

2) Transpalearctic, widely distributed from Europe to Far East (5 spp.): *Rophites canus*, *Halictus leucahenenus leucahenenus*, *H. quadricinctus*, *Lasioglossum albipes albipes*, and *L. calceatum*.

3) Transpalearctic-Oriental, widely distributed from Europe to Far East and southeastern Asia (2 spp.): *Ceylalictus variegatus* and *Lasioglossum villosulum*.

4) Europe to central Asia (33 spp.): *Systropha curvicornis*, *Pseudapis diversipes*, *P. femoralis*, *P. fugax*, *Nomioides minutissimus minutissimus*, *Halictus alfkenellus cedens*, *H. brunnescens*, *H. indefinitus*, *H. maculatus maculatus*, *H. mucoreus*, *H. nasica*, *L. obscuratum*, *H. pollinosa cariniventris*, *H. pulvereus*, *H. resurgens*, *H. seladonius*, *H. senilis*, *Lasioglossum buccale*, *L. clypeale*, *L. clypeiferellum*, *L. costulatum*, *L. denislucum*, *L. discum*, *L. griseolum*, *L. limbellum*, *L. lucidulum*, *L. mandibulare*, *L. marginatum*, *L. nitidiusculum*, *L. sexnotatulum*, *L. smeathmanellum*, and *L. xanthopus*.

5) Western to central Asia, nearly endemic in central Asia (42 spp.): *Dufourea paradoxa atrocoerulea*, *Nomioides gussakovskiji*, *N. ino*, *Halictus bucharicus*, *H. bulbiceps*, *H. compressus transvolgensis*, *H. duplocinctus*, *H. fuscicollis*, *H. mucidus*, *Lasioglossum niveocinctum*, *H. palustris*, *H. pjalmensis pjalmensis*, *H. pseudomucoreus*, *H. takuiricus*, *H. tibialis*, *H. transbaikalensis*, *L. acephalum*, *L. alanum*, *L. aprilinum*, *L. cingulatum*, *L. ciscapum*, *L. croceipes*, *L. equestre*, *L. fedtschenkoi*, *L. fulvitarse*, *L. hyalinipenne*, *L. kozlovi*, *L. laevinode*, *L. lebedevi*, *L. longirostre*, *L. melanopus*, *L. persicum*, *L. popovi*, *L. pseudonigripes*, *L. quadrinotatiforme*, *L. rhynchites*, *L. salinaecola*, *L. scutellare*, *L. subaenescens asiaticum*, *L. subequestre*, *L. tschardschuicum*, and *L. verae*.

6) Central Asia to Far East (2 spp.): *Halictus minor* and *Lasioglossum matianense pluto*.

7) Southern to central Asia. (1 sp.): *Lasioglossum sublaterale*.

The halictid fauna were mostly composed of Western to Central Asian elements (47.7 %), followed by the European to central Asian elements (37.5 %) in our suveyed area.

Many specimens belonging to Halictus (Seladonia), H. (Vestitohalictus), Lasioglossum (Dialictus), and L. (Hemihalictus) remain unidentified.

## Supplementary Material

Supplementary material 1 Specimens data Data type: Occurences
Brief description: The specimens data of halictid bees collected by Central Asian Expedition during 2000 to 2004 and 2012 to 2014.
File: oo_155910.xlsx Murao R., Tadauchi O.

XML Treatment for Rophitinae

XML Treatment for Dufourea paradoxa atrocoerulea

XML Treatment for Rophites (Rophitoides) canus

XML Treatment for Systropha (Systropha) curvicornis

XML Treatment for Nomiinae

XML Treatment for Pseudapis (Nomiapis) diversipes

XML Treatment for Pseudapis (Nomiapis) femoralis

XML Treatment for Pseudapis (Nomiapis) fugax

XML Treatment for Nomioidinae

XML Treatment for Ceylalictus (Ceylalictus) variegatus

XML Treatment for Nomioides gussakovskiji

XML Treatment for Nomioides ino

XML Treatment for Nomioides minutissimus minutissimus

XML Treatment for Halictinae

XML Treatment for Halictus (Argalictus) senilis

XML Treatment for Halictus (Argalictus) tibialis

XML Treatment for Halictus (Halictus) brunnescens

XML Treatment for Halictus (Halictus) duplocinctus

XML Treatment for Halictus (Halictus) quadricinctus

XML Treatment for Halictus (Hexataenites) resurgens

XML Treatment for Halictus (Monilapis) compressus transvolgensis

XML Treatment for Halictus (Mucoreohalictus) indefinitus

XML Treatment for Halictus (Mucoreohalictus) mucidus

XML Treatment for Halictus (Mucoreohalictus) mucoreus

XML Treatment for Halictus (Mucoreohalictus) pollinosus cariniventris

XML Treatment for Halictus (Mucoreohalictus) pseudomucoreus

XML Treatment for Halictus (Placidohalictus) bulbiceps

XML Treatment for Halictus (Placidohalictus) fuscicollis

XML Treatment for Halictus (Platyhalictus) alfkenellus cedens

XML Treatment for Halictus (Platyhalictus) minor

XML Treatment for Halictus (Platyhalictus) takuiricus

XML Treatment for Halictus (Protohalictus) bucharicus

XML Treatment for Halictus (Protohalictus) rubicundus

XML Treatment for Halictus (Seladonia) leucaheneus leucaheneus

XML Treatment for Halictus (Seladonia) pjalmensis pjalmensis

XML Treatment for Halictus (Seladonia) seladonius

XML Treatment for Halictus (Seladonia) transbaikalensis

XML Treatment for Halictus (Tytthalictus) maculatus maculatus

XML Treatment for Halictus (Tytthalictus) palustris

XML Treatment for Halictus (Vestitohalictus) nasica

XML Treatment for Halictus (Vestitohalictus) persephone

XML Treatment for Halictus (Vestitohalictus) pulvereus

XML Treatment for Lasioglossum (Dialictus) alanum

XML Treatment for Lasioglossum (Dialictus) fedtschenkoi

XML Treatment for Lasioglossum (Dialictus) smeathmanellum

XML Treatment for Lasioglossum (Hemihalictus) buccale

XML Treatment for Lasioglossum (Hemihalictus) ciscapum

XML Treatment for Lasioglossum (Hemihalictus) clypeare

XML Treatment for Lasioglossum (Hemihalictus) clypeiferellum

XML Treatment for Lasioglossum (Hemihalictus) croceipes

XML Treatment for Lasioglossum (Hemihalictus) denislucum

XML Treatment for Lasioglossum (Hemihalictus) griseolum

XML Treatment for Lasioglossum (Hemihalictus) laevinode

XML Treatment for Lasioglossum (Hemihalictus) limbellum

XML Treatment for Lasioglossum (Hemihalictus) longirostre

XML Treatment for Lasioglossum (Hemihalictus) lucidulum

XML Treatment for Lasioglossum (Hemihalictus) matianense pluto

XML Treatment for Lasioglossum (Hemihalictus) melanopus

XML Treatment for Lasioglossum (Hemihalictus) nitidiusculum

XML Treatment for Lasioglossum (Hemihalictus) persicum

XML Treatment for Lasioglossum (Hemihalictus) popovi

XML Treatment for Lasioglossum (Hemihalictus) pseudonigripes

XML Treatment for Lasioglossum (Hemihalictus) subaenescens asiaticum

XML Treatment for Lasioglossum (Hemihalictus) tschardschuicum

XML Treatment for Lasioglossum (Hemihalictus) villosulum

XML Treatment for Laioglossum (Lasioglossum) acephalum

XML Treatment for Lasioglossum (Lasioglossum) costulatum

XML Treatment for Lasioglossum (Lasioglossum) equestre

XML Treatment for Lasioglossum (Lasioglossum) fulvitarse

XML Treatment for Lasioglossum (Lasioglossum) lebedevi

XML Treatment for Lasioglossum (Lasioglossum) quadrinotatiforme

XML Treatment for Lasioglossum (Lasioglossum) sexnotatulum

XML Treatment for Lasioglossum (Lasioglossum) subequestre

XML Treatment for Lasioglossum (Lasioglossum) sublaterale

XML Treatment for Lasiolglossum (Lasioglossum) verae

XML Treatment for Lasioglossum (Lasioglossum) xanthopus

XML Treatment for Lasioglossum (Leuchalictus) discum

XML Treatment for Lasioglossum (Leuchalictus) leucozonium

XML Treatment for Lasioglossum (Leuchalictus) niveocinctum

XML Treatment for Lasioglossum (Leuchalictus) scutellare

XML Treatment for Lasioglossum (Leuchalictus) zonulum

XML Treatment for Lasioglossum (Sphecodogastra) albipes albipes

XML Treatment for Lasioglossum (Sphecodogastra) aprilinum

XML Treatment for Lasioglossum (Sphecodogastra) calceatum

XML Treatment for Lasioglossum (Sphecodogastra) cingulatum

XML Treatment for Lasioglossum (Sphecodogastra) hyalinipenne

XML Treatment for Lasioglossum (Sphecodogastra) obscuratum

XML Treatment for Lasioglossum (Sphecodogastra) rhynchites

XML Treatment for Lasioglossum kozlovi

XML Treatment for Lasioglossum mandibulare

XML Treatment for Lasioglossum marginatum

XML Treatment for Lasioglossum salinaecola

## Figures and Tables

**Figure 1a.**
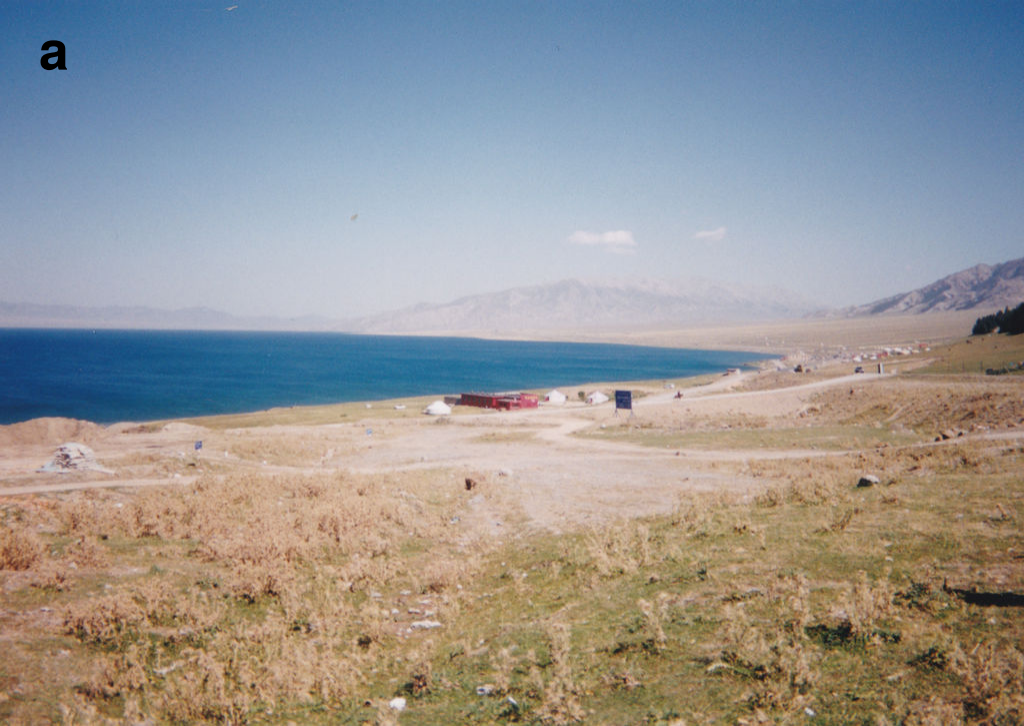
CN8: near Sayram Lake, Ili Prov., China

**Figure 1b.**
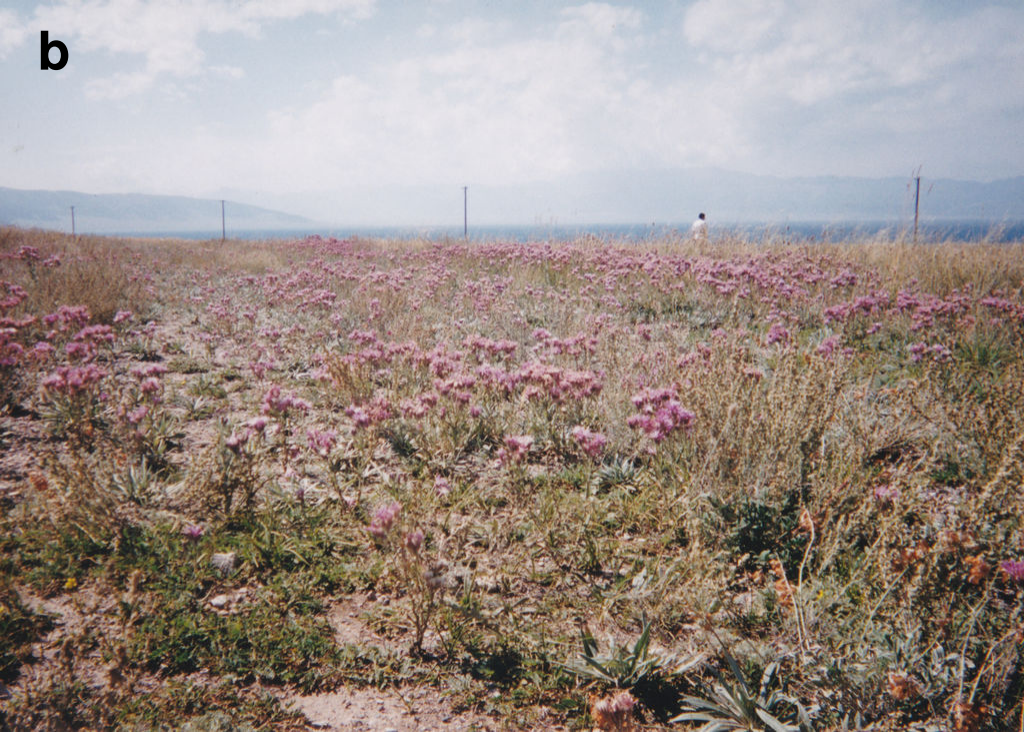
CN8: near Sayram Lake, Ili Prov., China

**Figure 1c.**
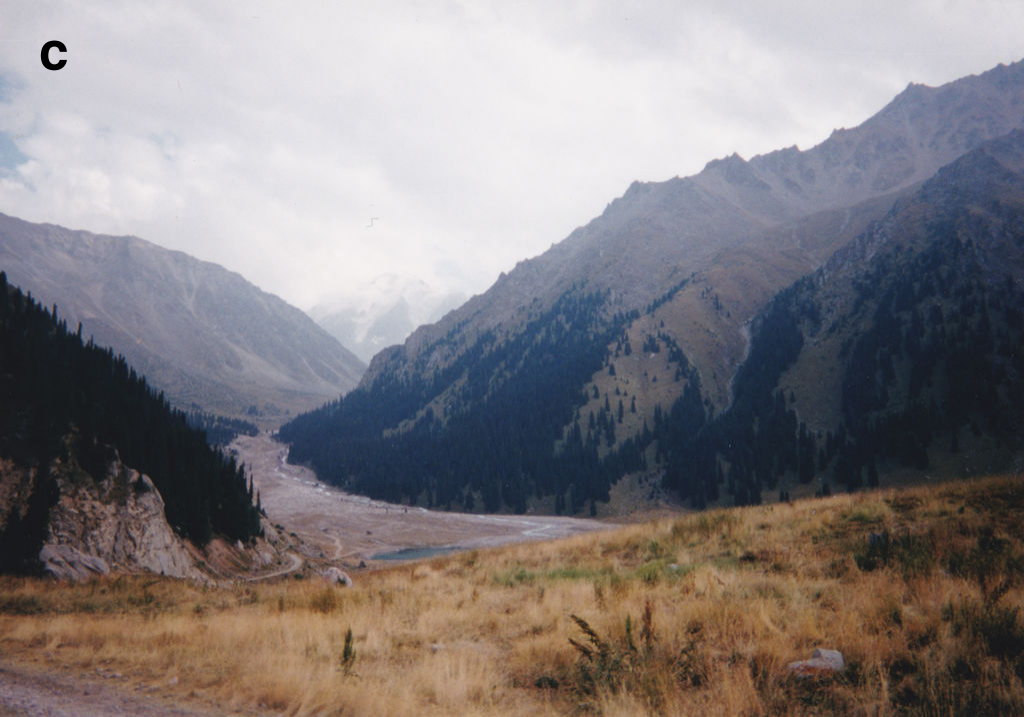
KZ13: Big Almaty Lake, Almaty Prov., Kazakhstan

**Figure 1d.**
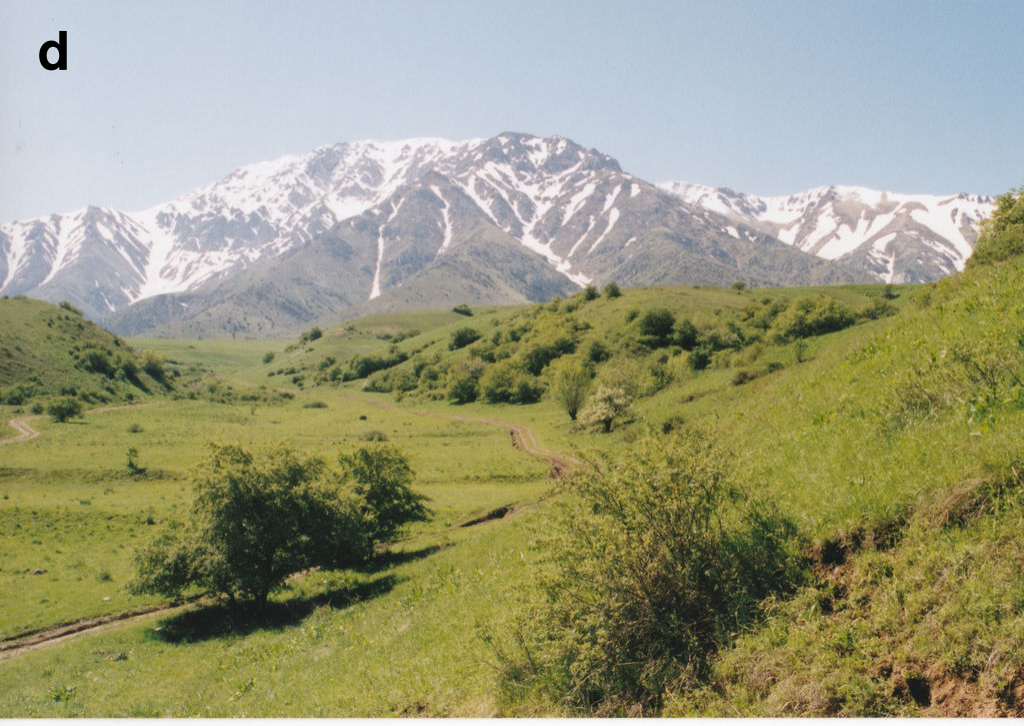
KZ37: Aksu Jabagly, South Kazakhstan Prov., Kazakhstan

**Figure 1e.**
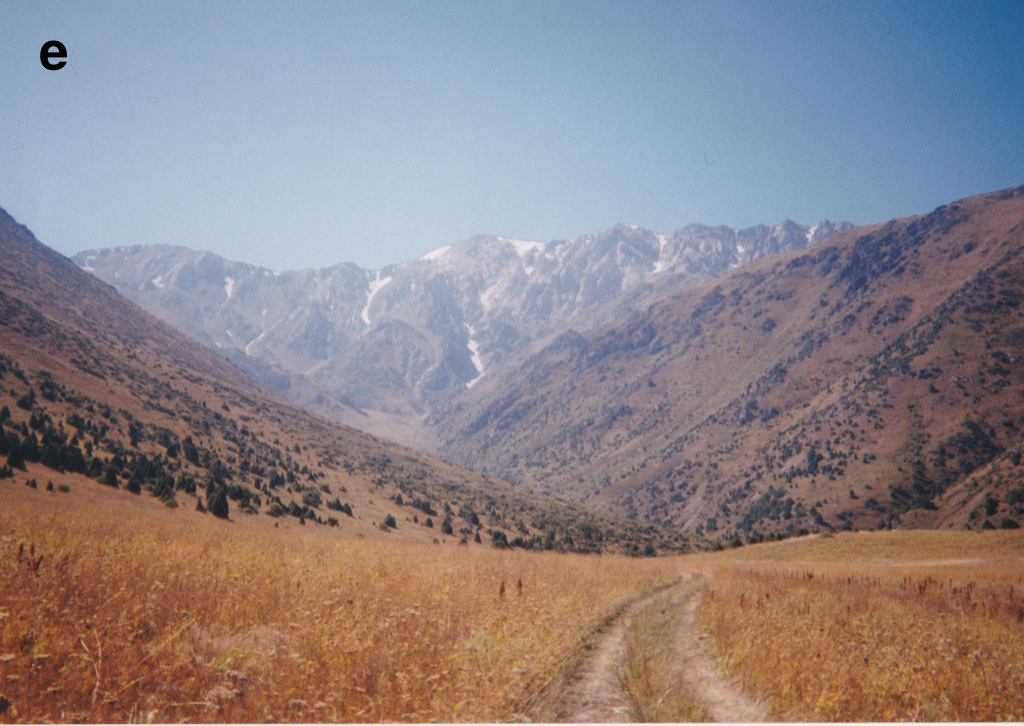
KZ37: Aksu Jabagly, South Kazakhstan Prov., Kazakhstan

**Figure 1f.**
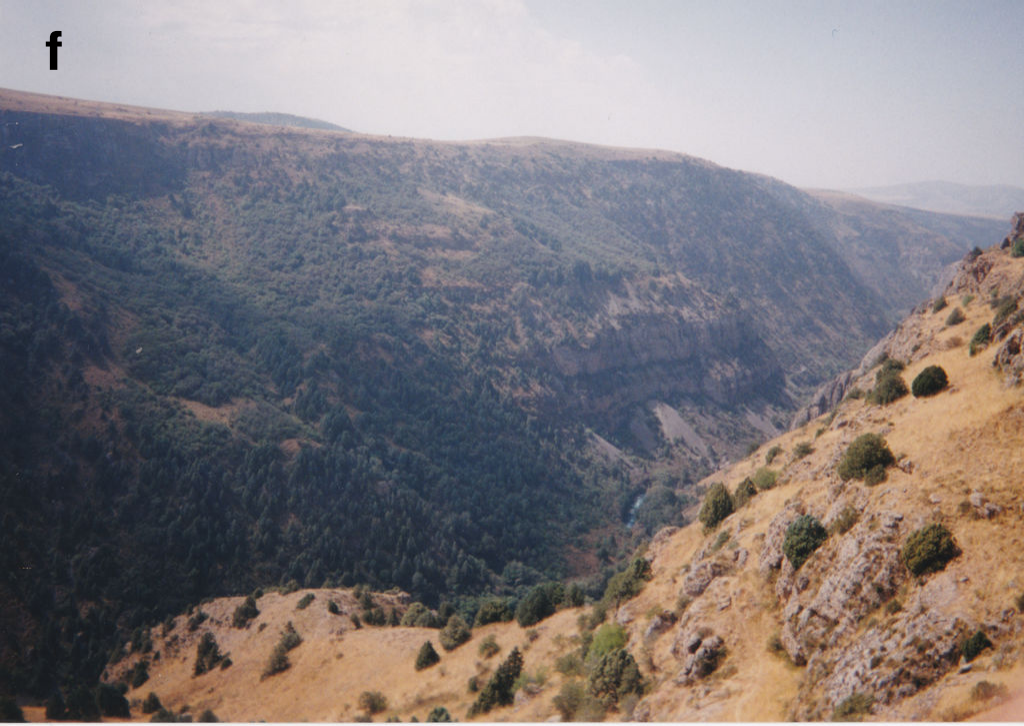
KZ38: Aksu valley, Jabagly, South Kazakhstan Prov., Kazakhstan

**Figure 2a.**
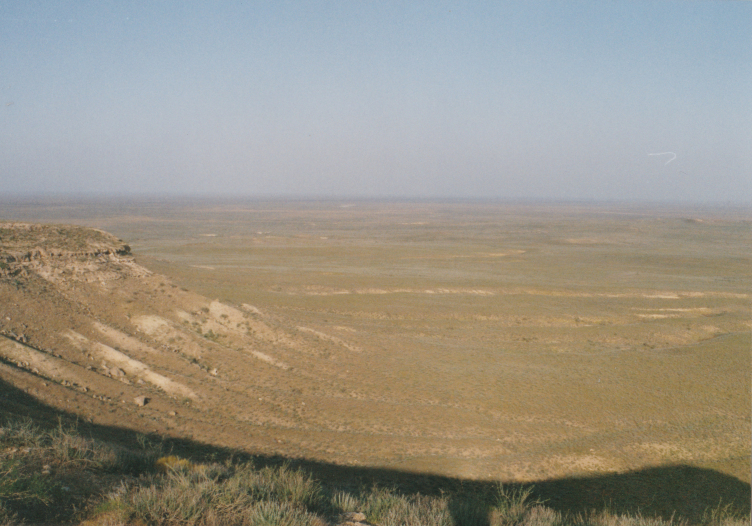
KZ60: Plain North of Karamola, Kyzylkum desert, South Kazakhstan Prov., Kazakhstan

**Figure 2b.**
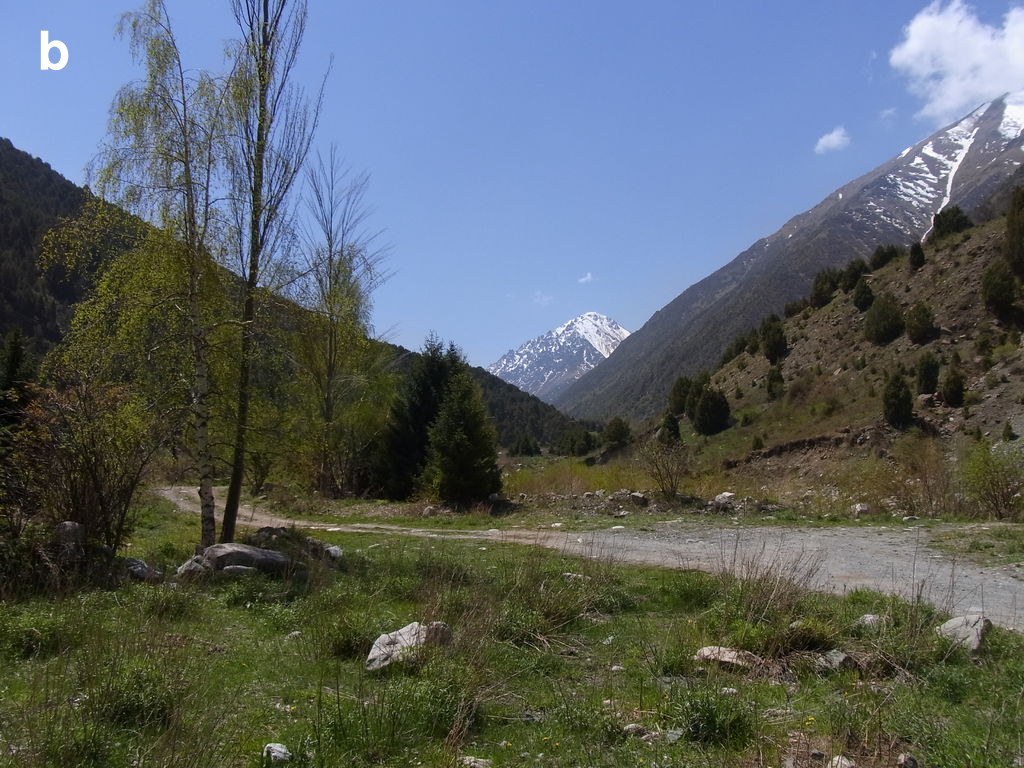
KG3: Ara Archa, Chuy Prov., Kyrgyzstan

**Figure 2c.**
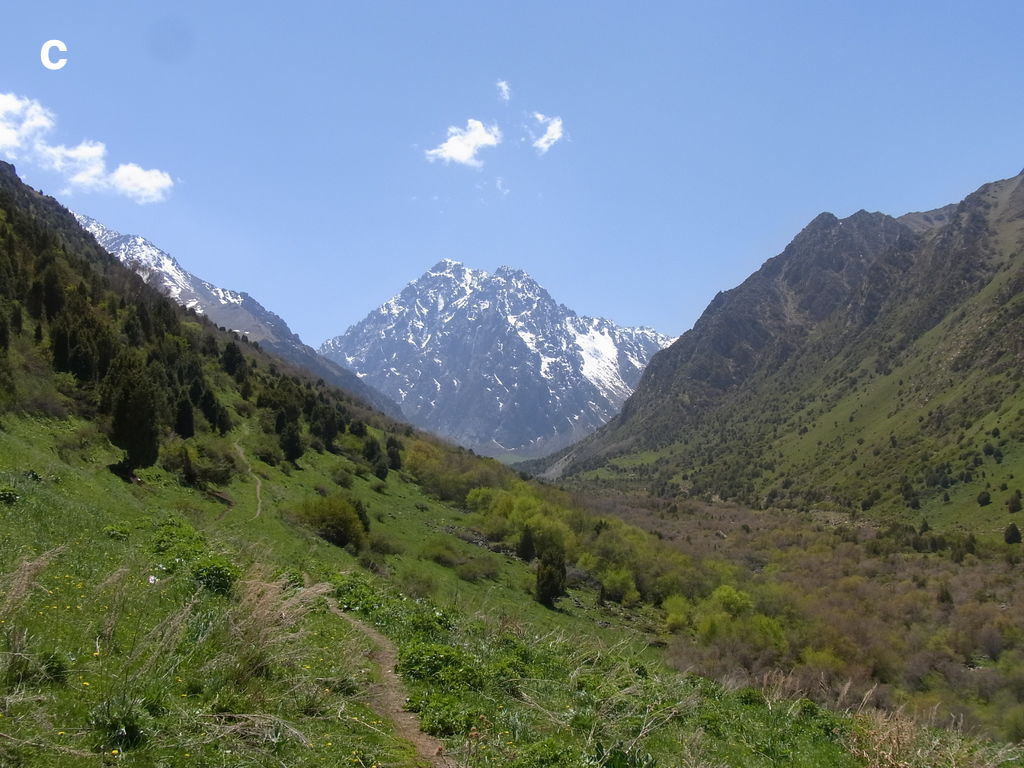
KG5: Issyk-Ata, Chuy Prov., Kyrgyzstan

**Figure 2d.**
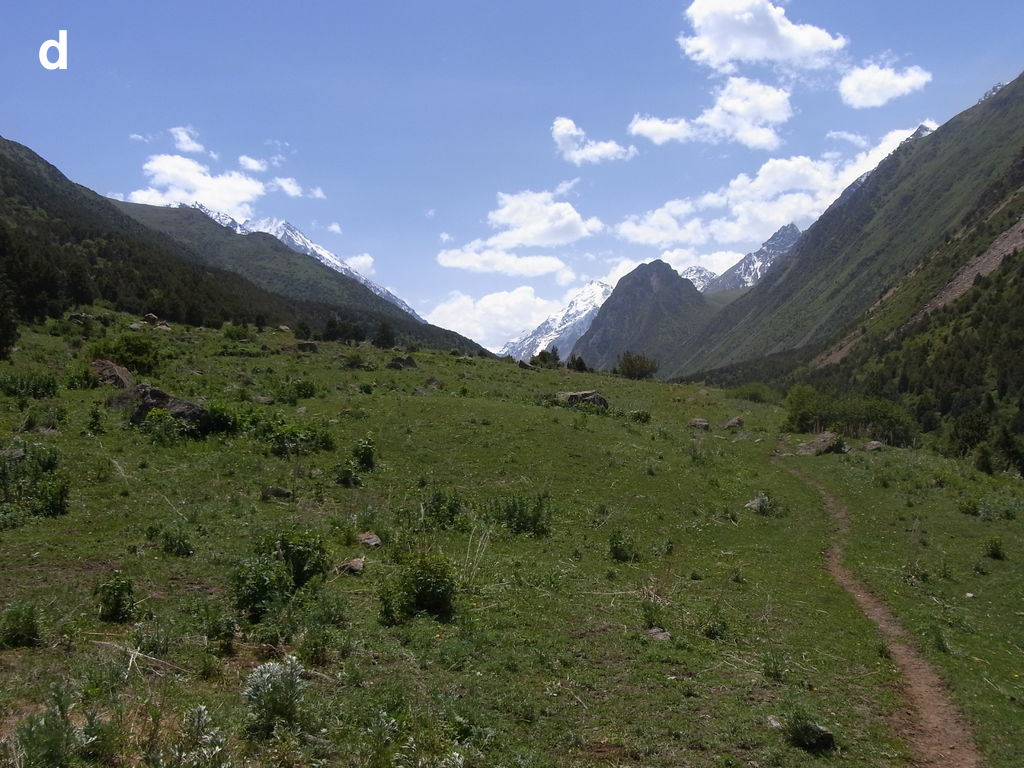
KG6: Koi Tash, Chuy Prov., Kyrgyzstan

**Figure 2e.**
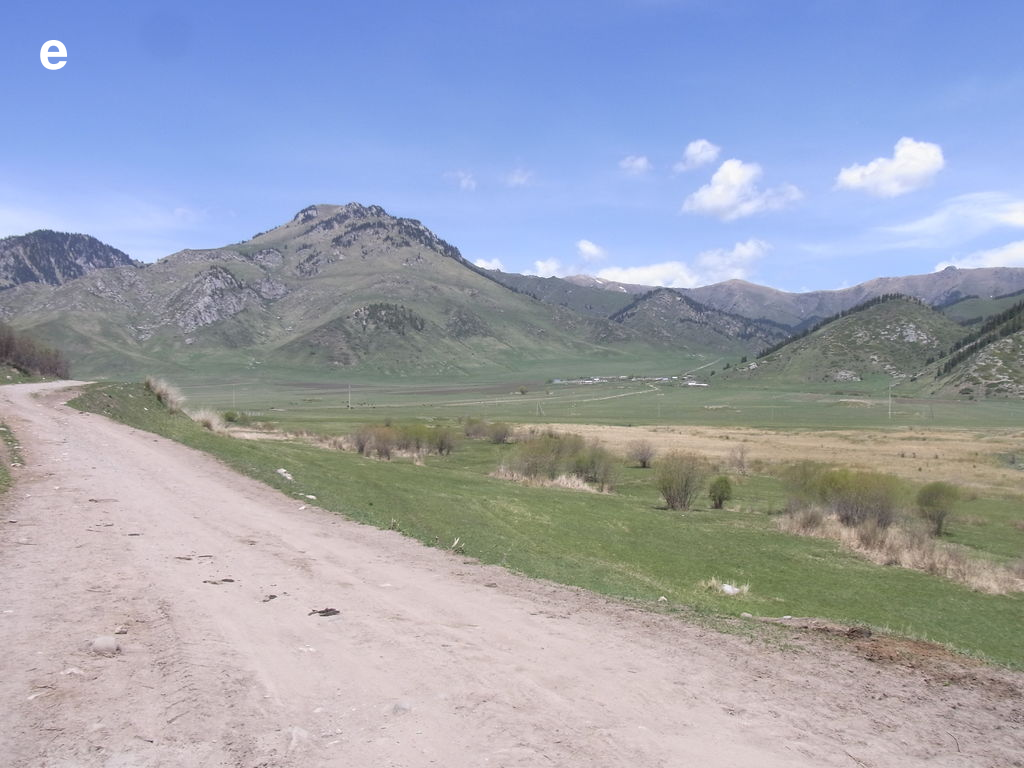
KG29: near San Tash, Issyk-Kul Prov., Kyrgyzstan

**Figure 2f.**
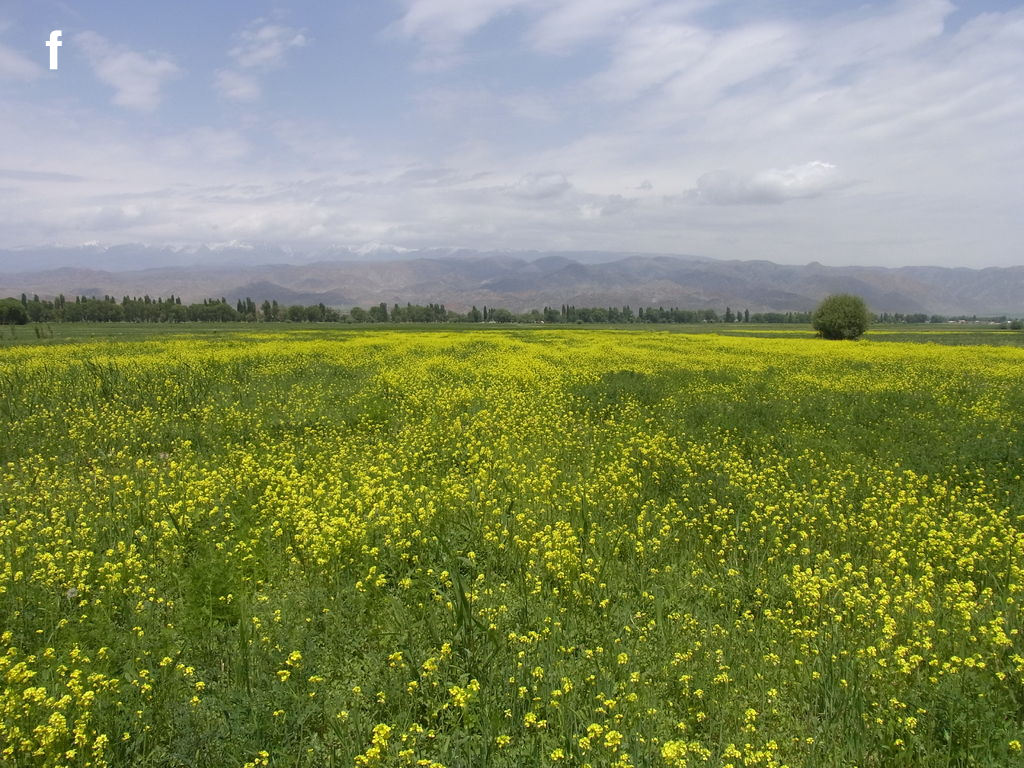
KG39: Ak-Kiya, Naryn Prov., Kyrgyzstan

**Figure 3a.**
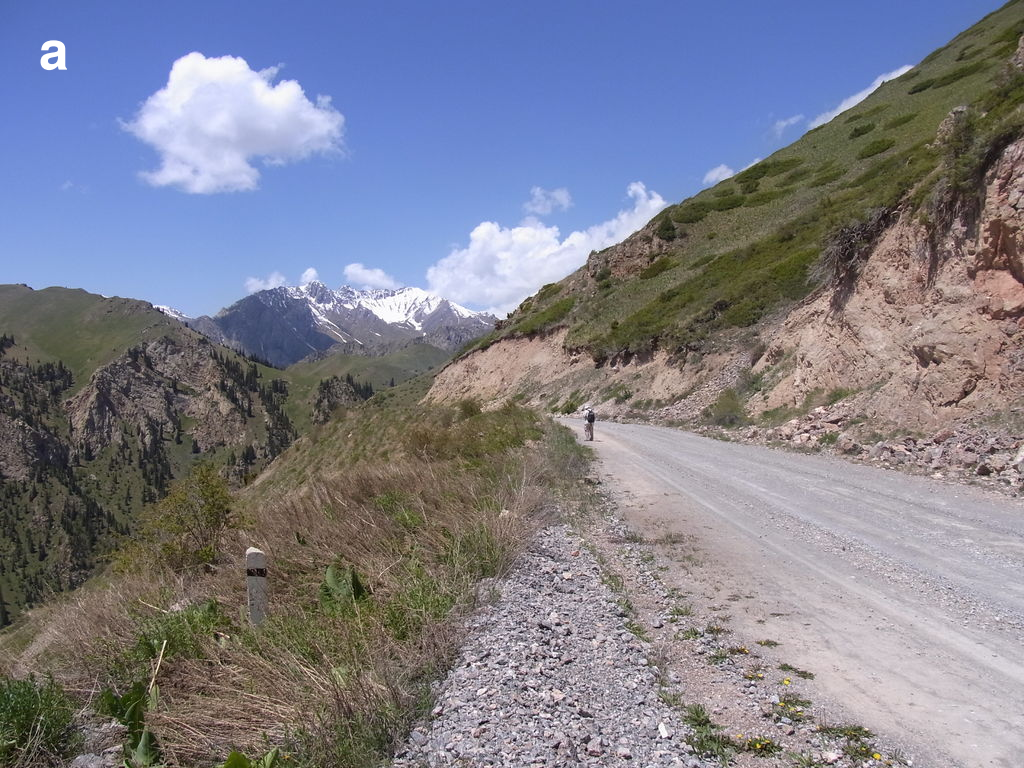
KG47: Moldo-Ashuu Pass, Naryn Prov.

**Figure 3b.**
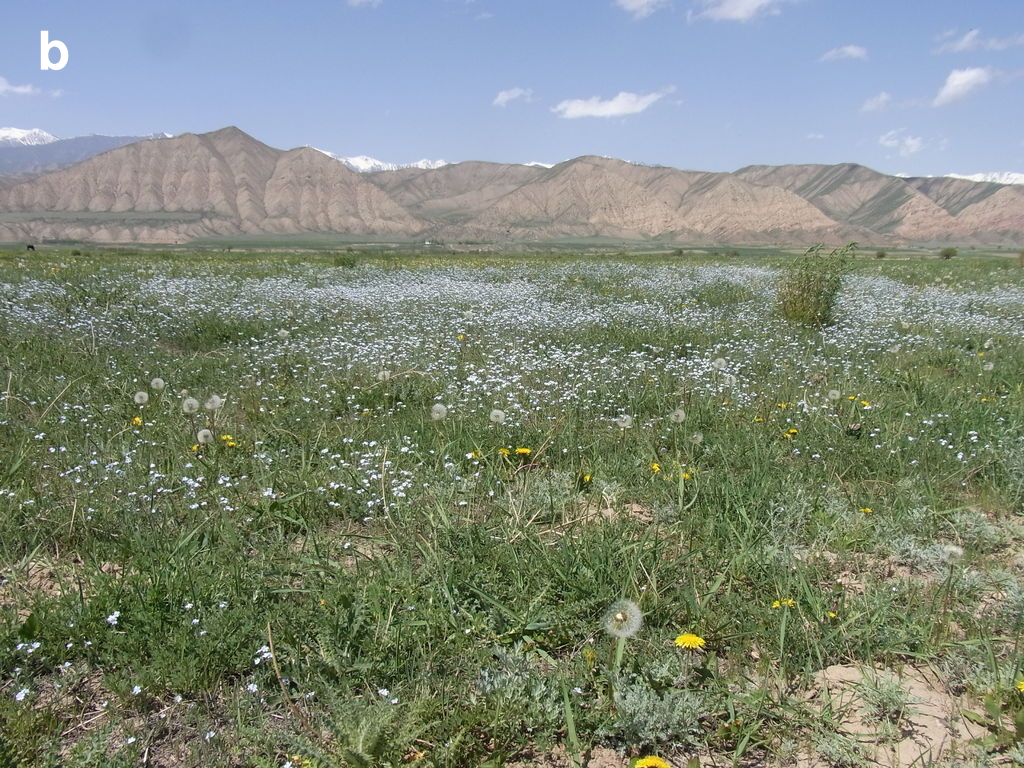
KG49: Naryn, Naryn Prov.

**Figure 3c.**
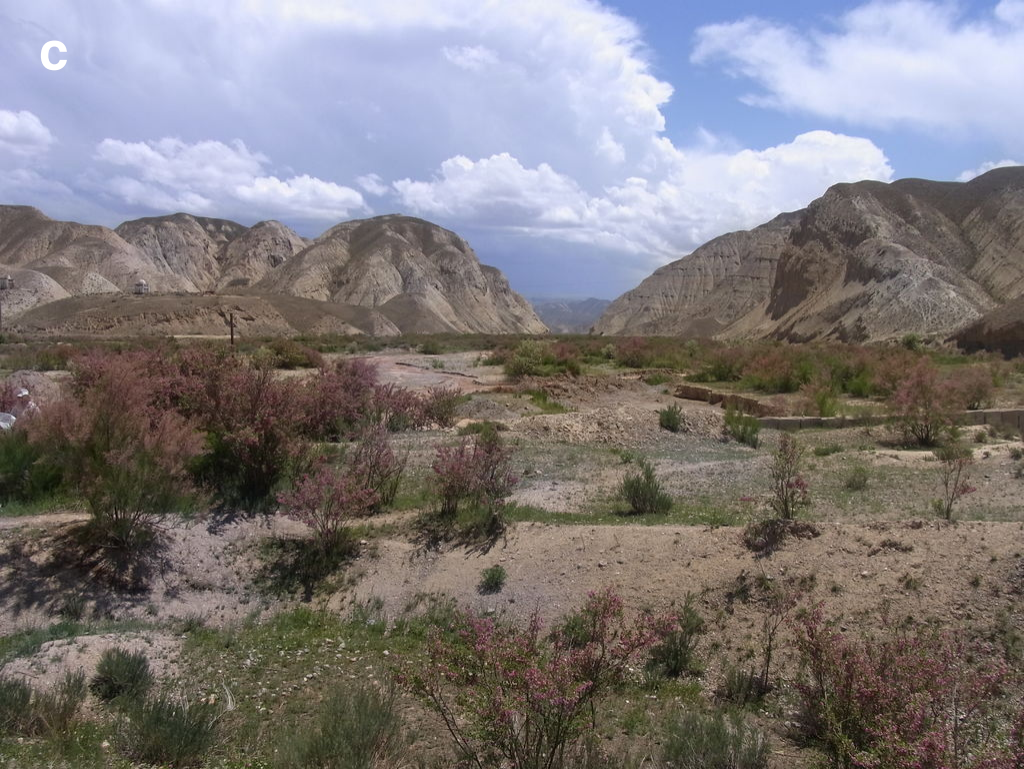
KG52: West of Naryn, Naryn Prov.

**Figure 3d.**
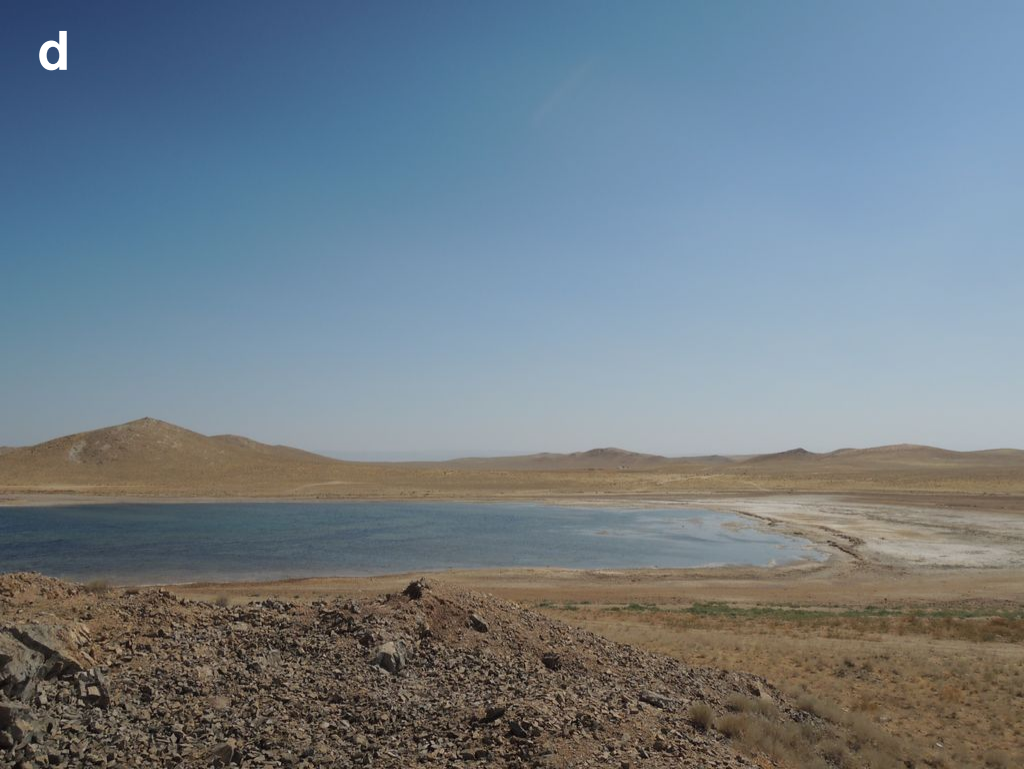
UZ1: Aydar Lake, Uzbekistan

**Figure 3e.**
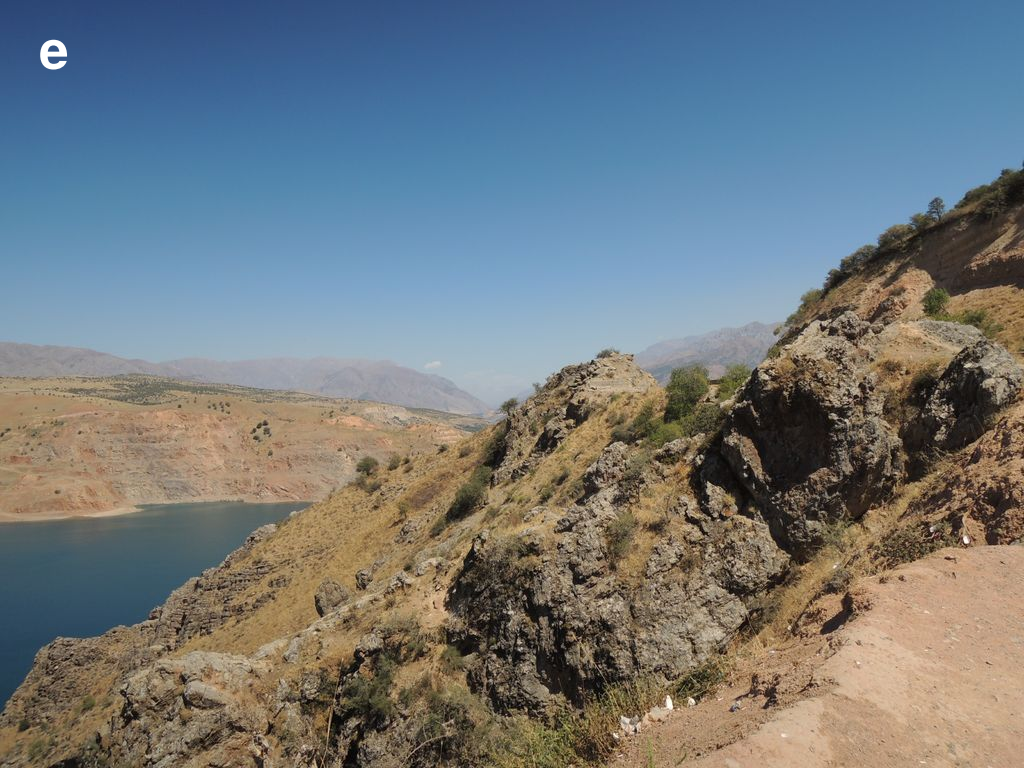
UZ3: Dalla Hovli, Northeast of Chirchik, Uzbekistan

**Figure 3f.**
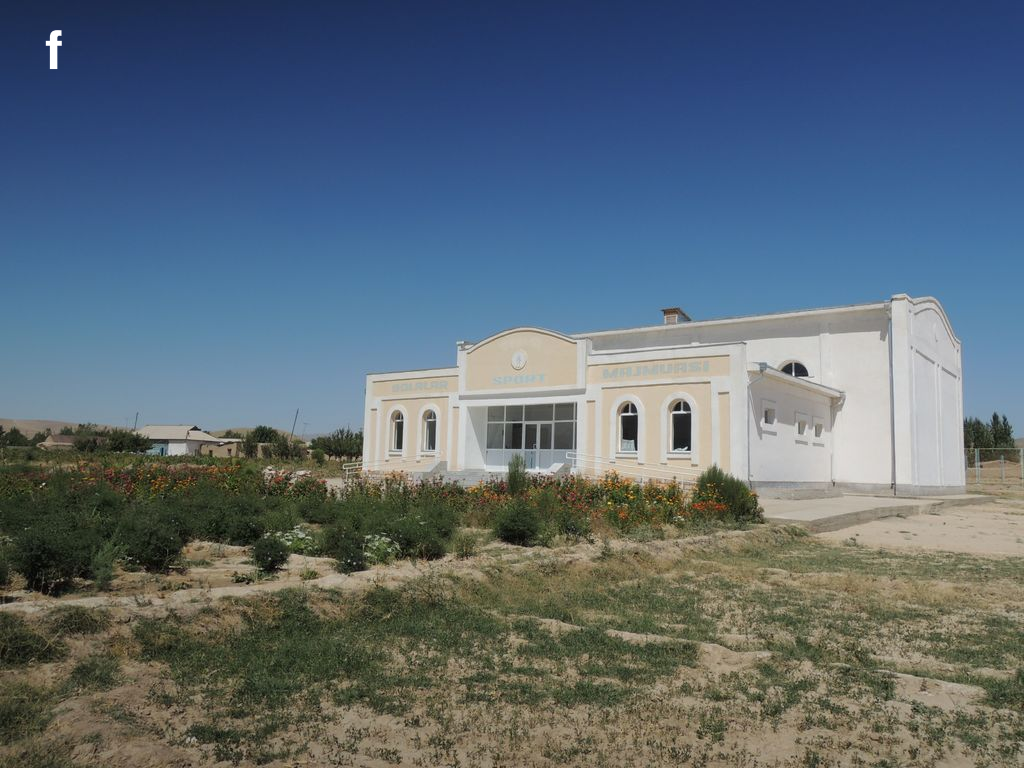
UZ7: Gushrabot, South of Aydar Lake, Uzbekistan

**Figure 4a.**
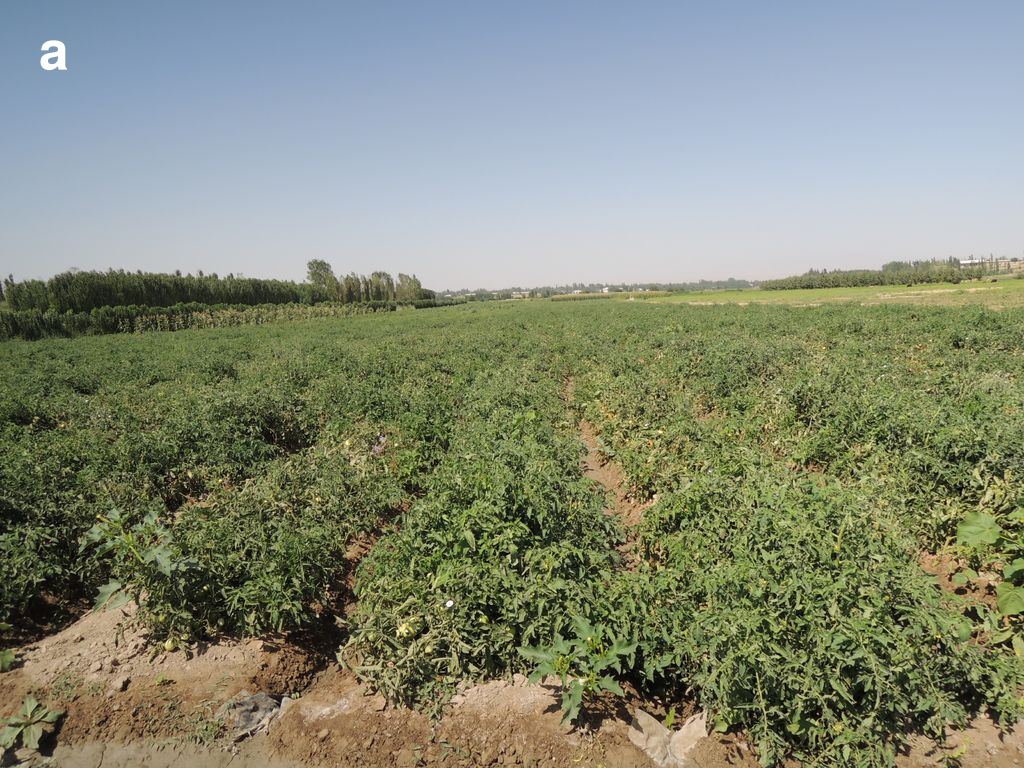
UZ8: Madaniyat village, East of Samarkand, Uzbekistan

**Figure 4b.**
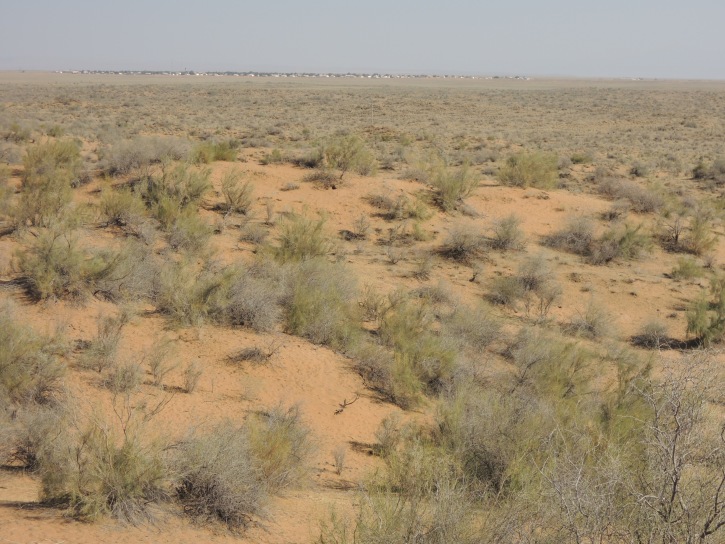
UZ12: South of Aydar Lake, Uzbekistan

**Figure 4c.**
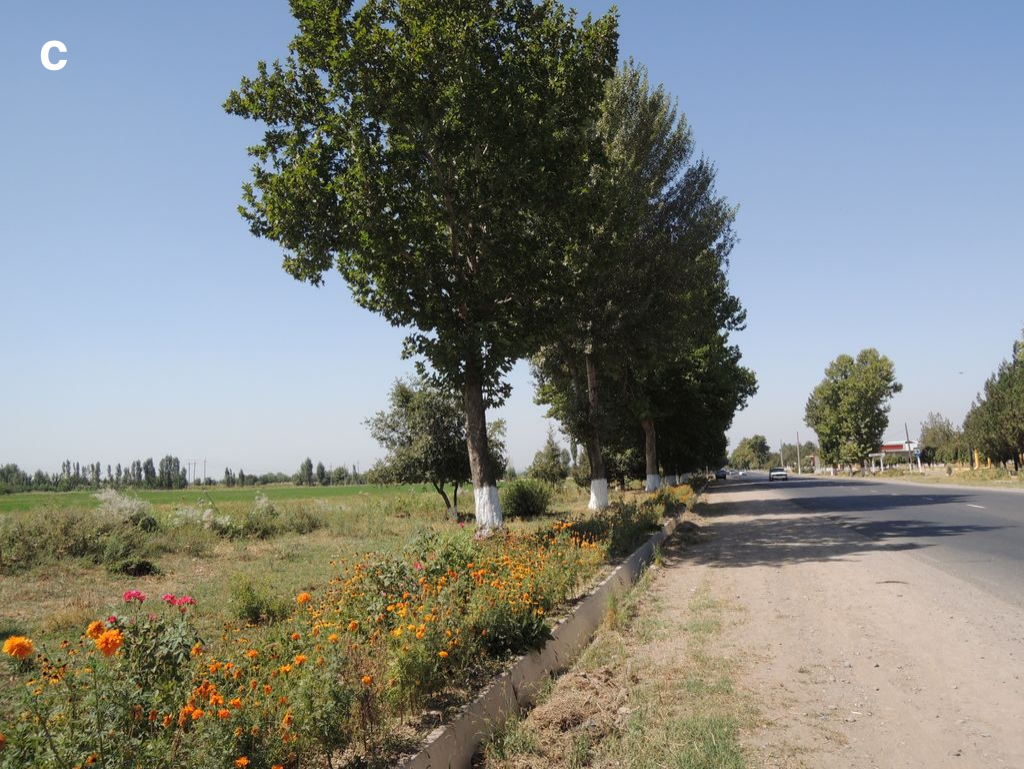
UZ13: Samarkand～Tashkent, Uzbekistan

**Figure 4d.**
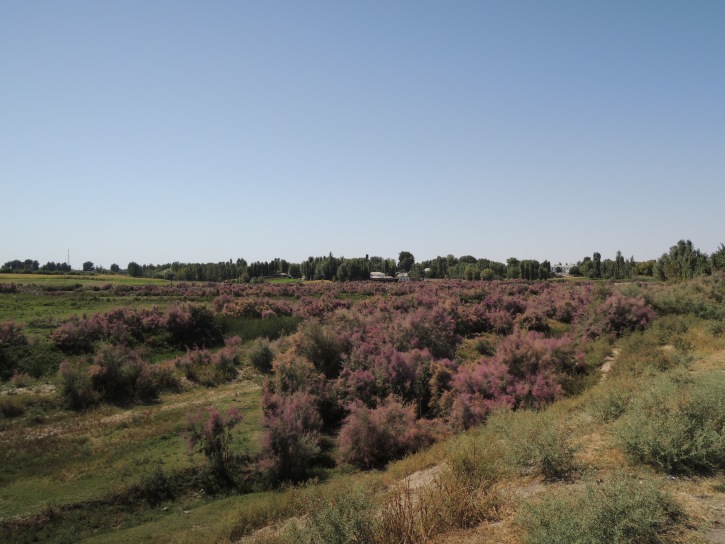
UZ15: Southwest of Yangiyo'l, South of Tashkent, Uzbekistan

**Figure 5a.**
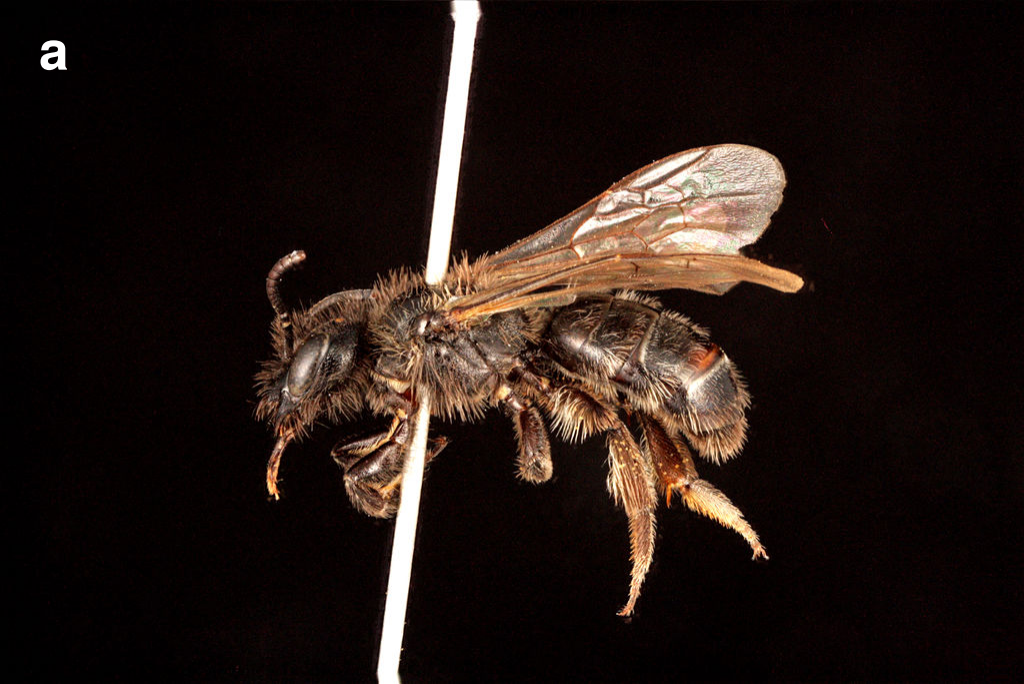
*Dufourea paradoxa atrocoerulea* (Morawitz, 1876)

**Figure 5b.**
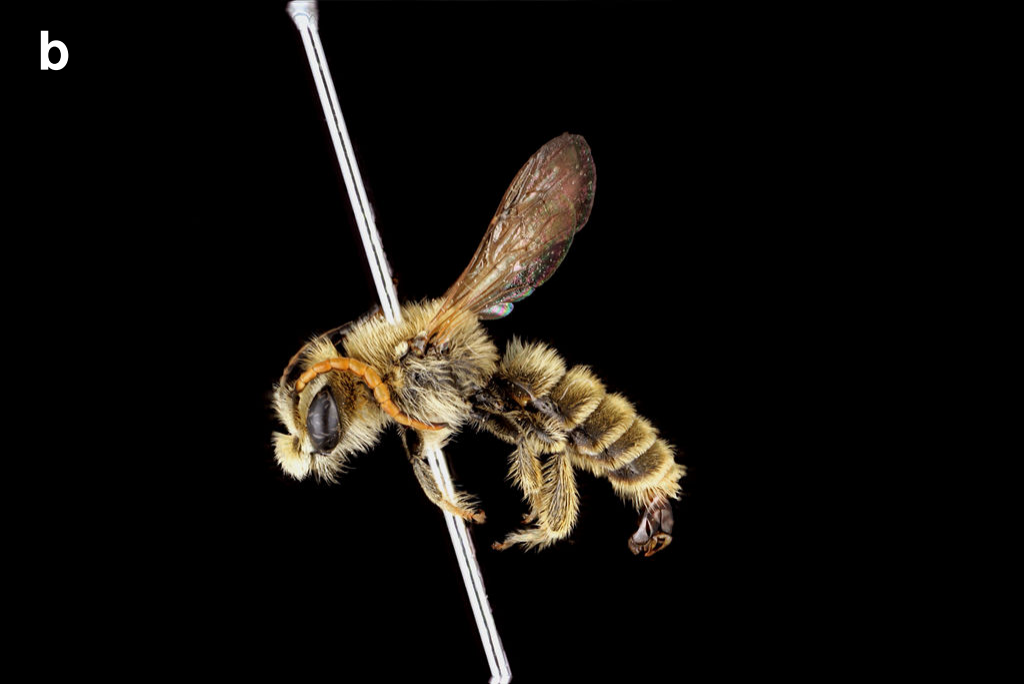
Rophites (Rophitoides) canus Eversmann, 1852

**Figure 5c.**
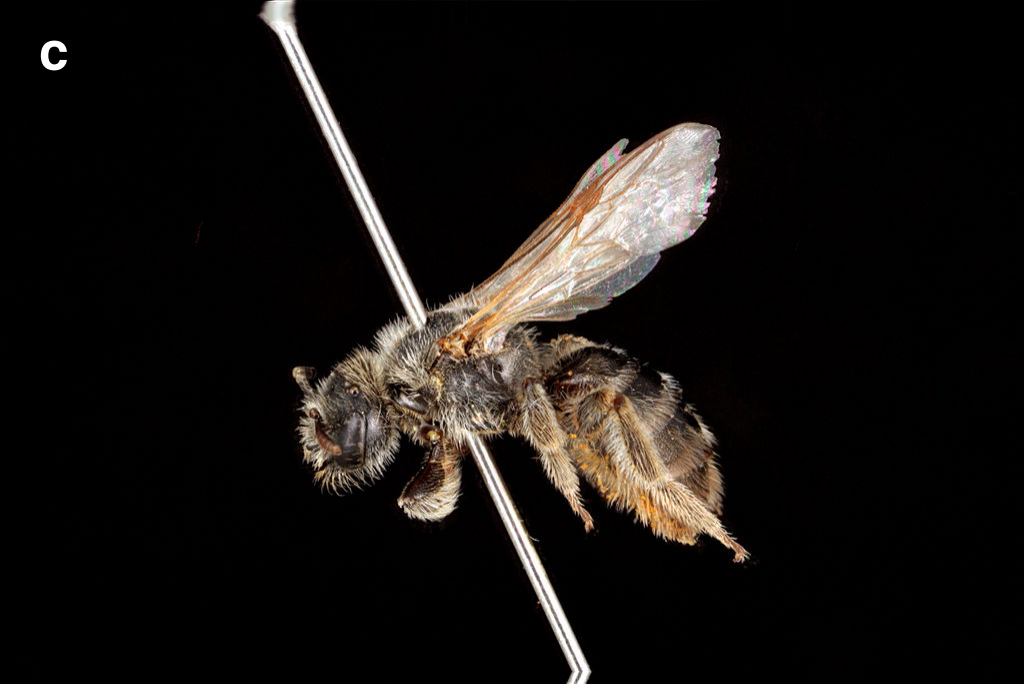
Systropha (Systropha) curvicornis (Scopoli, 1770)

**Figure 5d.**
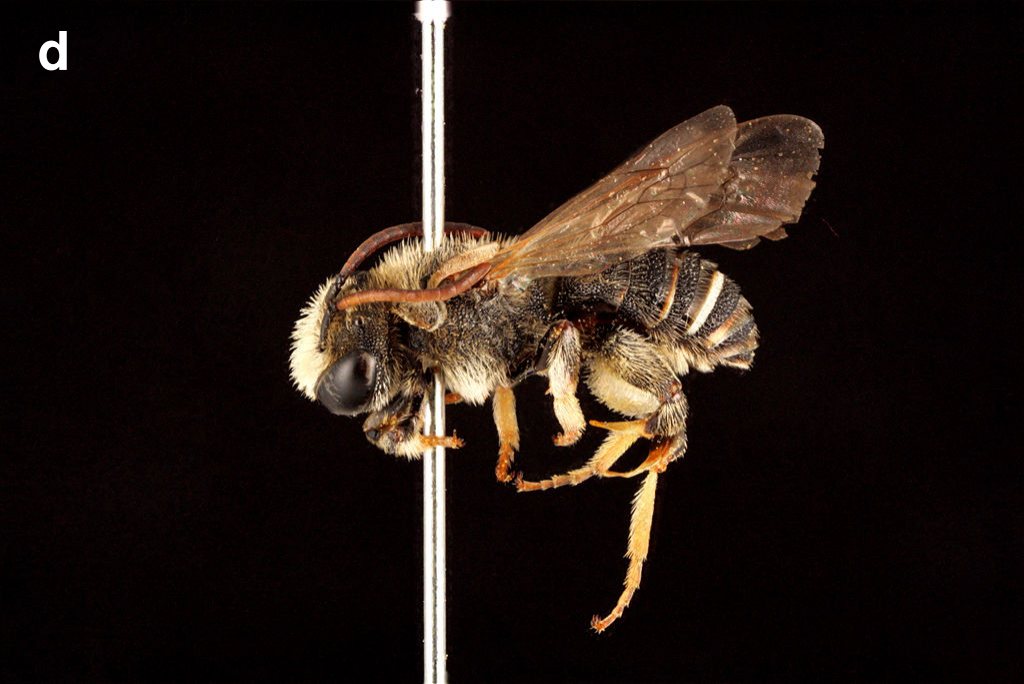
Pseudapis (Nomiapis) diversipes (Latreille, 1806)

**Figure 6a.**
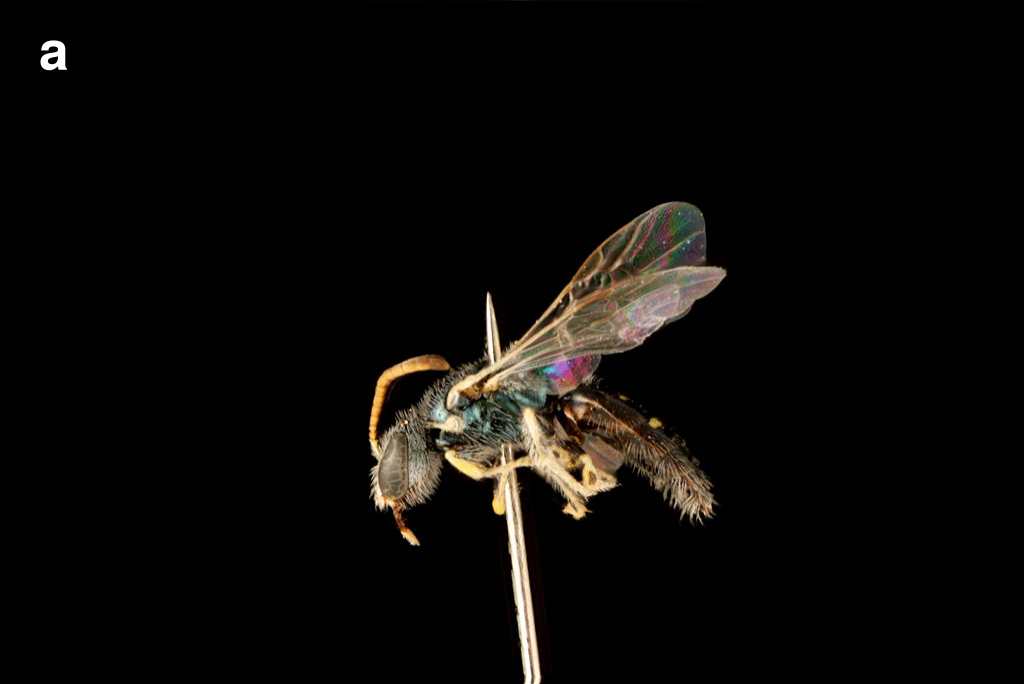
Ceylalictus (Ceylalictus) variegatus (Olivier, 1789)

**Figure 6b.**
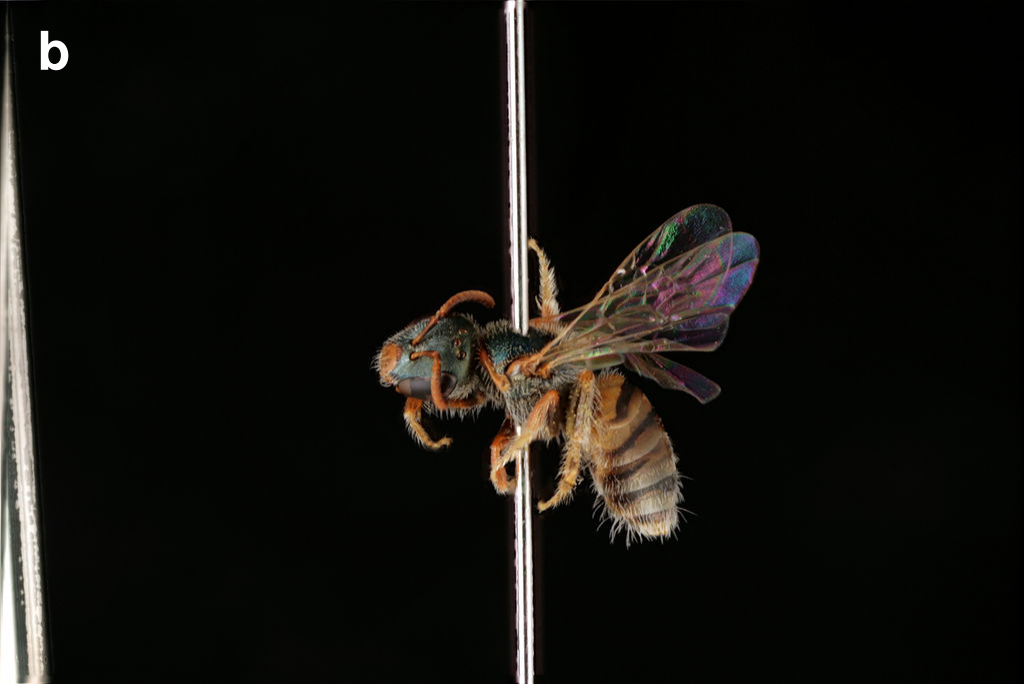
*Nomioides minutissimus minutissimus* (Rossi, 1790)

**Figure 6c.**
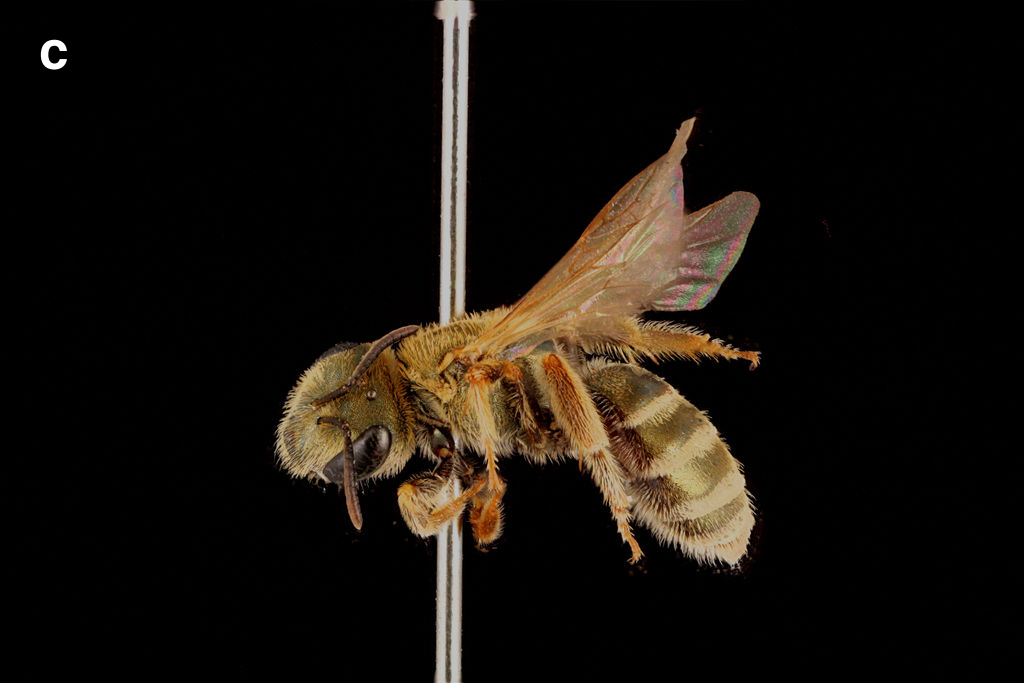
Halictus (Seladonia) pjalmensis pjalmensis Strand, 1909

**Figure 6d.**
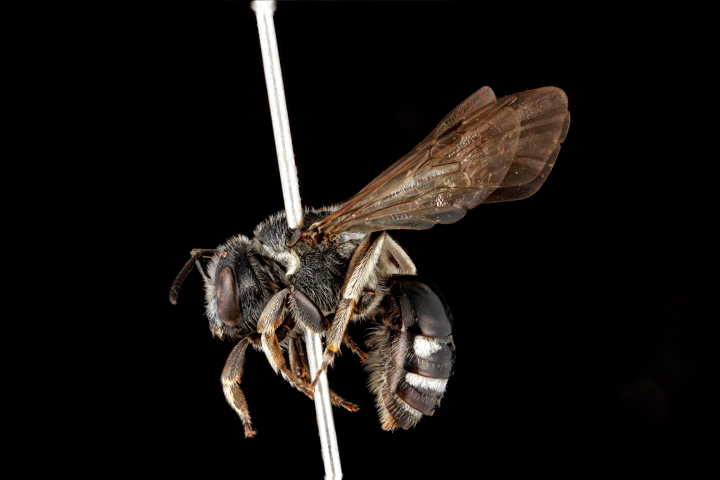
Lasioglossum (Lasioglossum) costulatum (Kriechbaumer, 1873).

**Table 1.** List of halictid bee species collected by Central Asian Expedition during 2000 to 2004 and 2012 to 2014.

Subfamily	Species	Country				Total
		Xinjiang Uyghur of China	Kazakhstan	Kyrgyzstan	Uzbekistan	
Rophitinae	Dufourea paradoxa atrocoerulea		2			2
Rophitinae	Rophites (Rophitoides) canus			13		13
Rophitinae	Systropha (Systropha) curvicornis	2				2
Nomiinae	Pseudapis (Nomiapis) diversipes	5	44	17	11	77
Nomiinae	Pseudapis (Nomiapis) femoralis	1		5		6
Nomiinae	Pseudapis (Nomiapis) fugax	11			21	32
Nomioidinae	Ceylalictus (Ceylalictus) variegatus	4	44	2	14	64
Nomioidinae	Nomioides gussakouskiji	7	3			10
Nomioidinae	Nomioides ino	7				7
Nomioidinae	Nomioides minutissimus minutissimus	187	4	9	3	203
Halictinae	Halictus (Argalictus) senilis	3	50			53
Halictinae	Halictus (Argalictus) tibialis		2			2
Halictinae	Halictus (Halictus) brunnescens	17	486	14	6	523
Halictinae	Halictus (Halictus) duplocinctus		19	1		20
Halictinae	Halictus (Halictus) quadricinctus	4	6	44		54
Halictinae	Halictus (Hexataenites) resurgens	1	167	9	51	228
Halictinae	Halictus (Monilapis) compressus transvolgensis	1	201	159		361
Halictinae	Halictus (Mucoreohalictus) indefinitus	1	21			22
Halictinae	Halictus (Mucoreohalictus) mucidus		133			133
Halictinae	Halictus (Mucoreohalictus) mucoreus	4	217	83	2	306
Halictinae	Halictus (Mucoreohalictus) pollinosus cariniventris	1	3			4
Halictinae	Halictus (Mucoreohalictus) pseudomucoreus			1		1
Halictinae	Halictus (Placidohalictus) bulbiceps	2				2
Halictinae	Halictus (Placidohalictus) fuscicollis	1				1
Halictinae	Halictus (Platyhalictus) alfkenellus cedens			2		2
Halictinae	Halictus (Platyhalictus) minor		74	1		75
Halictinae	Halictus (Platyhalictus) takuiricus			2		2
Halictinae	Halictus (Protohalictus) bucharicus		31			31
Halictinae	Halictus (Protohalictus) rubicundus		1	7		8
Halictinae	Halictus (Seladonia) leucaheneus leucaheneus	1	4			5
Halictinae	Halictus (Seladonia) pjalmensis pjalmensis	2	305	45		352
Halictinae	Halictus (Seladonia) seladonius		137	63		200
Halictinae	Halictus (Seladonia) transbaikalensis		2			2
Halictinae	Halictus (Tytthalictus) maculatus maculatus			2		2
Halictinae	Halictus (Tytthalictus) palustris	4	65	117		186
Halictinae	Halictus (Vestitohalictus) nasica		11			11
Halictinae	Halictus (Vestitohalictus) persephone		2			2
Halictinae	Halictus (Vestitohalictus) pulvereus	144	16			160
Halictinae	Lasioglossum (Dialictus) alanum		9	1		10
Halictinae	Lasioglossum (Dialictus) fedtschenkoi		4			4
Halictinae	Lasioglossum (Dialictus) smeathmanellum		6	1		7
Halictinae	Lasioglossum (Hemihalictus) buccale		3			3
Halictinae	Lasioglossum (Hemihalictus) ciscapum		4			4
Halictinae	Lasioglossum (Hemihalictus) clypeare		1			1
Halictinae	Lasioglossum (Hemihalictus) clypeiferellum		78	7		85
Halictinae	Lasioglossum (Hemihalictus) croceipes		141	1		142
Halictinae	Lasioglossum (Hemihalictus) denislucum		4	4		8
Halictinae	Lasioglossum (Hemihalictus) griseolum		4	1		5
Halictinae	Lasioglossum (Hemihalictus) laevinode		28			28
Halictinae	Lasioglossum (Hemihalictus) limbellum limbellum		4			4
Halictinae	Lasioglossum (Hemihalictus) longirostre		32	15		47
Halictinae	Lasioglossum (Hemihalictus) lucidulum			20		20
Halictinae	Lasioglossum (Hemihalictus) matianense pluto		54	170		224
Halictinae	Lasioglossum (Hemihalictus) melanopus		2	28		30
Halictinae	Lasioglossum (Hemihalictus) nitidiusculum		1			1
Halictinae	Lasioglossum (Hemihalictus) persicum		2			2
Halictinae	Lasioglossum (Hemihalictus) popovi		11			11
Halictinae	Lasioglossum (Hemihalictus) pseudonigripes		81	92		173
Halictinae	Lasioglossum (Hemihalictus) subaenescens asiaticum		26	1		27
Halictinae	Lasioglossum (Hemihalictus) tschardschuicum		1			1
Halictinae	Lasioglossum (Hemihalictus) villosulum		2			2
Halictinae	Lasioglossum (Lasioglossum) acephalum		16			16
Halictinae	Lasioglossum (Lasioglossum) costulatum		25	1		26
Halictinae	Lasioglossum (Lasioglossum) equestre		42	14		56
Halictinae	Lasioglossum (Lasioglossum) fulvitarse		2	34		36
Halictinae	Lasioglossum (Lasioglossum) lebedevi		1			1
Halictinae	Lasioglossum (Lasioglossum) quadrinotatiforme		217	5		222
Halictinae	Lasioglossum (Lasioglossum) sexnotatulum			21		21
Halictinae	Lasioglossum (Lasioglossum) subequestre		22			22
Halictinae	Lasioglossum (Lasioglossum) sublaterale		2			2
Halictinae	Lasioglossum (Lasioglossum) verae	2				2
Halictinae	Lasioglossum (Lasioglossum) xanthopus		204	74		278
Halictinae	Lasioglossum (Leuchalictus) discum	1	155	44	18	218
Halictinae	Lasioglossum (Leuchalictus) leucozonium	5	42	15	1	63
Halictinae	Lasioglossum (Leuchalictus) niveocinctum	2				2
Halictinae	Lasioglossum (Leuchalictus) scutellare	2	82	1		85
Halictinae	Lasioglossum (Leuchalictus) zonulum		7			7
Halictinae	Lasioglossum (Sphecodogastra) albipes albipes		7	2		9
Halictinae	Lasioglossum (Sphecodogastra) aprilinum	1	205			206
Halictinae	Lasioglossum (Sphecodogastra) calceatum	3	132	394		529
Halictinae	Lasioglossum (Sphecodogastra) cingulatum		7	1		8
Halictinae	Lasioglossum (Sphecodogastra) hyalinipennis		15	79		94
Halictinae	Lasioglossum (Sphecodogastra) obscuratum		30	1		31
Halictinae	Lasioglossum (Sphecodogastra) rhynchites		34	115		149
Halictinae	Lasioglossum kozlovi	9				9
Halictinae	Lasioglossum mandibulare	1	30			31
Halictinae	Lasioglossum marginatum		9799	458		10257
Halictinae	Lasioglossum salinaecola		1			1
Total	88 spp.	436	13625	2196	127	16384

## References

[B3673147] Ascher J. S., Pickering J. Discover Life bee species and world checklist (Hymenoptera: Apoidea: Anthophila). http://www.discoverlife.org./mp/20q?guide=Apoidea_species.

[B3673156] Astafurova Yu. V. (2004). A new species of the genus *Pseudapis* W.F. Kirby (Hymenoptera: Halictidae) from Tajikistan. Trudy Russkogo Entomologicheskogo Obschestva.

[B3680513] Astafurova Yu. V., Pesenko Yu. A. (2005). Contributions to the halictid fauna of the Eastern Palaearctic Region: subfamily Nomiinae (Hymenoptera: Halictidae). Far Eastern Entomologist.

[B3673176] Blüthgen P. (1923). Beiträge zur Systematik der Bienengattung *Halictus* Latr.. Konowia.

[B3673166] Blüthgen P. (1923). Beiträge zur Kenntnis der Bienengattung *Halictus* Latr.. Archiv für Naturgeschichte.

[B3673186] Blüthgen P. (1923). Beiträge zur Systematik der Bienengattung *Sphecodes* Latr.. Deutsche Entomologische Zeitschrift.

[B3673196] Blüthgen P. (1924). Beiträge zur Systematik der Bienengattung *Halictus* Latr. (Hym.). II. Die Gruppe des *Hal.
albipes* F.. Konowia.

[B3673206] Blüthgen P. (1925). Beiträge zur Kenntnis der Bienengattung *Halictus* Latr. II.. Archiv für Naturgeschichte.

[B3673216] Blüthgen P. (1929). Neue turkestanische *Halictus*-Arten (Hym. Apidae).. Konowia.

[B3673226] Blüthgen P. (1931). Beitrage zur Synonymie der Bienengattung *Halictus* Latr. III.. Mitteilungen aus dem Zoologischen Museum in Berlin.

[B3673236] Blüthgen P. (1933). Neue Arten aus der Gattung *Nomioides* Schenck (Hym. Apidae
Halictinae
Nomioidini C. B.). Memorie della Societa Entomologica Italiana.

[B3673246] Blüthgen P. (1933). Neue paläarktische *Halictus*-Arten (Hym., Apidae). I. Grüne Binden *Halictus*.. Deutsch Entomologische Zeitschrift.

[B3673256] Blüthgen P. (1934). Nachtrag zur Monographie der Bienengattung *Nomioides* Schck. (Hym., Apidae, Halictinae.).. Stettiner entomologische Zeitung.

[B3673266] Blüthgen P. (1934). Neue turkestanische *Halictus*-Arten. II. (Hym. Apidae).. Konowia.

[B3673276] Blüthgen P. (1936). Neue paläarktische Binden-*Halictus* (Hym. Apidae).. Mitteilungen aus dem Zoologischen Museum in Berlin.

[B3673286] Blüthgen P. (1955). The Halictinae (Hymen., Apoidea) of Israel. I. Genus *Halictus* (subgenera *Halictus* s. str. and *Thrincohalictus*). Bulletin of the Research Council of Israel Ser. B.

[B3673296] Danforth B. N., Brady S. G., Spipes S. D., Pearson A. (2004). Single-copy nuclear genes recover Cretaceous-Age divergences in bees. Systematic Biology.

[B3673306] Ebmer A. W. (1972). Neue westpaläarktische Halictidae (Halictinae, Apoidea). Mitteilungen aus dem Zoologischen Museum in Berlin.

[B3673316] Ebmer A. W. (1980). Asiatische Halictidae (Apoidea, Hymenoptera). Linzer Biologische Beiträge.

[B3673326] Ebmer A. W. (1995). Asiatische Halictidae, 3. Die Arten-gruppe der *Lasioglossum* carinate-*Evylaeus* (Insecta: Hymenoptera: Apoidea: Halictidae: Halictinae). Linzer Biologische Beiträge.

[B3673336] Ebmer A. W. (1997). Asiatische Halictidae, 6. *Lasioglossum* carinaless-*Evylaeus*: Ergänzungen zu den Artengruppe von *L.
nitidiusculum* und *L.
punctatissimum* s.l., sowie die Artengruppe des *L.
marginellum* (Insecta: Hymenoptera: Apoidea: Halictidae: Halictinae). Linzer Biologische Beiträge.

[B3673346] Ebmer A. W. (2005). Zur Bienenfauna der Mongolei Die Arten der Gattungen *Halictus* Latr. und *Lasioglossum* Curt. (Insecta: Hymenoptera: Apoidea: Halictidae: Halictinae) Ergänzungen und Korrekturen.. Linzer Biologische Beiträge.

[B3673356] Ebmer A. W., Sakagami S. F. (1985). Taxonomic notes on the Palaearctic species of the *Lasioglossum
nitidiusculum* group, with description of *L.
allodalum* sp. nov. (Hymenoptera, Halictidae). Kontyû.

[B3673366] Handlirsch A. (1888). Die Bienengattung Nomioides Schenck.. Verhandlungen der zoologish-botanischen Gesellschaft in Wien (Abhandlungen).

[B3673376] Kuhlmann M. (2009). Bees of the genus *Colletes* (Hymenoptera, Colletidae) from central Asia collected by the Kyushu University Expeditions. Esakia.

[B3673386] Maeta Y., Miyanaga R., Kitamura K. (2003). Ecological studies on the wild bee fauna at Mt. Sanbe in Shimane Prefecture, Japan (Hymenoptera, Apoidea). New Entomologist.

[B3673396] Michener C. D. (1974). *The Social Behavior of the Bees*.

[B3673414] Michener C. D. (1979). Biogeography of the bees. Annals of the Missouri Botanical Garden.

[B3673424] Michener C. D. (2007). *The Bees of the World, 2nd ed.*.

[B3673433] Mitai K. (2012). A new species of the genus *Sphecodes* (Hymenoptera: Halictidae) from Kazakhstan collected by the Kyushu University Expeditions. Esakia.

[B3673443] Mitai K., Tadauchi O. (2008). The genus *Nomada* (Hymenoptera, Apidae) from Kazakhstan and Kyrgyzstan collected by the Kyushu University Expedition (1). Esakia.

[B3673453] Miyanaga R., Tadauchi O., Murao R. (2006). Notes on the nest architecture of *Halictus
senilis* (Eversmann) in southeast Kazakhstan (Hymenoptera, Halictidae). Esakia.

[B3673463] Morawitz F. (1876). Pchely (Mellifera). II. Andrenidae / Puteshestvie v Turkestan … A.P.Fedchenko [Bees (Mellifera) / Travel to Turkestan by … A.P. Fedchenko. No. 13, t. 2. Zoological Researches. Part 5, book 2]. Izvestia Imeratorskogo Obshchestva Lyubitelei Estestvoznania, Etnographii i Antropologii.

[B3673473] Morawitz F (1880). Ein Beitrag zur Bienen-Fauna Mittel-Asiens. Bulletin de l'Académie impériale des sciences de St.-PétersbourgBulletin de l'Académie impériale des sciences de St.-Pétersbourg.

[B3673483] Morawitz F (1893). Supplement zur Bienenfauna Turkestans. Horae Societatis Entomologicae Rossica.

[B3673493] Morawitz F (1894). Beitrag zur Bienenfauna Turkmeniens. Horae Societatis Entomologicae Rossica.

[B3673503] Murao R., Tadauchi O., Miyanaga R. (2015). The bee tribe Anthidiini (Hymenoptera, Megachilidae) collected from central Asia. Japanese Journal of Systematic Entomology.

[B3673513] Niu Z. Q., Wu Y. R., Huang D. W. (2005). A taxonomic study on the four genera of the subfamily Rophitinae from China (Hymenoptera: Halictidae). The Raffles Bulletin of Zoology.

[B3673523] Niu Z. Q., Zhu C. D., Zhang Y. Z., Wu Y. R., Huang D. W. (2007). A taxonimic study of the subgenus
Vestitohalictus of the genus *Halictus* (Hymenoptera, Halictidae, Halictinae) from China. Acta Zootaxonomica Sinica.

[B3673534] Pallas P. S. (1773). *Reise durch verschiedene Provinzen des Russischen Reiches, zweiter Theil, Erstes Buch vom Jahr 1770*.

[B3673543] Pèrez J (1903). Espèces nouvelles de Mellifères. Extrait des Pròces-Verbaux des sèances de la Société Linnéenne de Bordeaux.

[B3673553] Pesenko Yu. A. (1979). Novyi vid pchely roda *Nomioides* Schenck (Hymenoptera, Halictidae) iz Srednei Azii [A new species of the bee genus *Nomioides* Schenck (Hymenoptera, Halictidae) from the Middle Asia]. Trudy Vsesoyuznogo Entomologicheskogo Obshchestva.

[B3673573] Pesenko Yu. A. (1983). *Fauna of the USSR (n. s., no. 129). Hymenopterous insects. Vol. XVII, No. 1. Halictid bees (Halictidae). The tribe Nomioidini (in amount of the Palaearctic Region)*.

[B3673606] Pesenko Yu. A., Pesenko Yu. A. (1984). Systematics of bees of the genus *Halictus* Latreille (Hymenoptera, Halictidae) with description of 7th and 8th metasomal stema of males: subgenus
Platyhalictus. *Systematic and Ecology of Bees*.

[B3673582] Pesenko Yu. A., Korotyaev B. A. (1984). The bees of the genus *Halictus* Latreille sensu stricto (Hymenoptera, Halictidae) of Mongolia and northwestem China, with a review of publications on the Halictini of this region and with a revision of the subgenus
Prohalictus of the World fauna. *Insects of Mongolia*.

[B3673596] Pesenko Yu. A. (1984). A subgeneric classification of bees of the genus *Halictus* Latreille sensu stricto (Hymenoptera, Halictidae). Entomologicheskoe Obozrenie.

[B3673630] Pesenko Yu. A., Pesenko Yu. A. (1985). Systematics of bees of the genus *Halictus* Latreille (Hymenoptera, Halictidae) with description of 7th and 8th metasomal sterna of males: subgenus
Monilapis Cockerell. *Systematic and Ecology of Bees*.

[B3673644] Pesenko Yu. A., Pesenko Yu. A. (1986). An annotated key to the Palaearctic species of bees of the genus *Lasioglossum* sensu stricto (Hymenoptera, Halictidae) for females, with description of new subgenera and species. *Systematics of Hymenopterous Insects*.

[B3673658] Pesenko Yu. A. (1999). Phylogeny and classification of the family Halictidae revised (Hymenoptera: Apoidea). Journal of the Kansas Entomological Society.

[B3673678] Pesenko Yu. A. (2005). New data on the taxonomy and distribution of the Palaearctic halictids: genus *Halictus* Latreille (Hymenoptera: Halictidae). Entomofauna.

[B3673668] Pesenko Yu. A. (2005). Contributions to the halictid fauna of the Eastern Palaearctic Region: subfamily Nomioidinae (Hymenoptera: Halictidae). Far Eastern Entomologist.

[B3782334] Pesenko Yu. A. (2005). Contributions to the halictid fauna of the Eastern Palaearctic Region: genus *Halictus* Latreille (Hymenoptera: Halictidae, Halictinae). Far Eastern Entomologist.

[B3673688] Pesenko Yu. A. (2006). Contributions to the halictid fauna of the Eastern Palaearctic Region: genus *Seladonia* (Hymenoptera: Halictidae, Halictinae). Esakia.

[B3673698] Pesenko Yu. A., Astafurova Yu. A. (2006). Contributions to the halictid fauna of the Eastern Palaearctic Region: subfamily Rophitinae (Hymenoptera: Halictidae). Entomofauna.

[B3673717] Pesenko Yu. A., Wu Y. (1997). Chinese bees of the genus *Halictus* s. str. with descriptions of a new species and a new subspecies (Hymenoptera: Halictidae). Acta Entomologica Sinica.

[B3673708] Pesenko Yu. A., Banaszak J., Radchenko V. G., Cierzniak T. (2000). *Bees of the Family Halictidae (Excluding Sphecodes) of Poland: Taxonomy, Ecology, Bionomics*.

[B3673727] Plateaux-Quénu C. (1962). Biology of *Halictus
marginatus* Brullé. Journal of Apicultural Research.

[B3673737] Popov V. B. (1934). The bee fauna of Kokchetav district of northern Kazakhstan. Trudy Akademija Nauk SSSR, Kazachstankaja Baza.

[B3673747] Popov V. B. (1935). Contributions to the bee fauna of Tajikistan. Travaux de la Filiale de l'Academie des Sciences de l'URSS in Tadjikistan.

[B3673757] Popov V. B. (1949). Notes on the bee fauna (Hymenoptera, Apoidea) of Tajikistan. Trudy Zoologicheskova Instituta, Akademii Nauk SSSR.

[B3673777] Popov V. B. (1952). The bee fauna (Hymenoptera, Apoidea) of southwestern Turkmenistan and its landscape distribution. Trudy Zoologicheskova Instituta, Akademii Nauk SSSR.

[B3673787] Popov V. B. (1956). New and little-known bees (Hymenoptera, Apoidea) of Middle Asia. Entomologicheskoe Obozrenie.

[B3673797] Popov V. B. (1958). Zoogeographical peculiarities of the central Asian species of the genus *Halictoides* (Hymenoptera, Halictidae). Doklady Akademii Nauk Tadzhikskoe SSR.

[B3673807] Radoszkowski O. (1893). Fauna hyménoptèrologique transcaspienne. Horae Societatis Entomologicae Rossica.

[B3673817] Sakagami S. F., Fukuda H. (1973). Wild bee survey at the campus of Hokkaido University.. Journal of the Faculty of Science, Hokkaido University, Series VI, Zoology.

[B3673827] Schwarz M. P., Richards M. H., Danforth B. N. (2007). Changing paradigms in insect social evolution: insights from halictine and allodapine bees. Anuual Review of Entomology.

[B3673837] Shebl M. A., Tadauchi O. (2009). The genus *Andrena* from Kazakhstan and Kyrgyzstan (Hymenoptera, Andrenidae) (3). Esakia.

[B3673847] Strand E. (1909). Die palaarktischen *Halictus*-Arten des kgl. Zoologischen Museums zu Berlin, z. T. nach Bestimmungen von J. D. Alfken. Archiv für Naturgeschichte.

[B3673857] Tadauchi O. (2005). Field studies on wild bee fauna and pollination biology for combating desertification and planting campaigns in Asian arid areas: A report for the year 2000 to 2004. Esakia.

[B3673867] Tadauchi O. (2006). The genus *Andrena* from Kazakhstan and Kyrgyzstan collected by the Kyushu University Expedition (Hymenoptera, Andrenidae). Esakia.

[B3673877] Tadauchi O. (2008). The genus *Andrena* from Kazakhstan and Kyrgyzstan (Hymenoptera, Andrenidae) (2). Esakia.

[B3680524] Tadauchi O., Murao R. Entomology Database, BeeCAsia. http://konchudb.agr.agr.kyushu-u.ac.jp/beecasia/index-e.html.

[B3673887] Tadauchi O., Miyanaga R., Dawut A. (2005). A new species belonging to the subgenus
Euandrena of the genus *Andrena* from Xinjiang Uygur, China with notes on nest structure (Hymenoptera, Andrenidae). Esakia.

[B3673897] Vachal J (1902). *Halictus* nouveaux ou litigieux de la collection Radoszkovski (Hymenoptera, Apidae). *R*usskoe Entomologicheskoe Obozrenie.

[B3673907] Warncke K. (1976). Zur Systematik und Verbreitung der Bienengattung *Nomia* Latr. in der Westpaläarktis und dem turkestanischen Becken (Hymenoptera, Apoidea). Reichenbachia.

[B3673917] Williams P. H. (2011). Bumblebees collected by the Kyushu University Expeditions to Central Asia (Hymenoptera, Apidae, genus *Bombus*). Esakia.

[B3673927] Wu Y., Huang D. S., Han Y., Zhang X. (1985). The insect fauna of the Mt Tuomuer areas in Tianshan, Apoidea. Biota of Tuomuer region, Tianshan.

